# Supplementary material for: Helical supramolecular polymers with rationally designed binding sites for chiral guest recognition
Source: Nat Commun. 2020 May 8;11:2311. doi: 10.1038/s41467-020-16127-6 (PMC7210886; doi:10.1038/s41467-020-16127-6)
Supplement: Supplementary file 1 — Supplementary Information [file 41467_2020_16127_MOESM1_ESM.pdf]

Supplementary Information

Helical supramolecular polymers with rationally designed binding sites for chiral guest recognition

Salikolimi *et al.*

Supplementary Methods

General and Materials

General. Dynamic light scattering (DLS) measurement was performed with a Malvern model Zetasizer Nano ZSP analyzer at 25 °C using a quartz cuvette with a 10-mm optical path length. Fourier transform infrared (FT-IR) spectra were measured on a JASCO model FT/IR-4100 spectrometer equipped with a home-made temperature controller. UV/Vis and circular dichroism (CD) spectra were measured on a JASCO model J-1500 spectropolarimeter equipped with a JASCO model PTC-510 Peltier-type temperature controller. Atomic-force microscopy (AFM) images were taken on an Oxford Instruments model Cypher system. Scanning electron microscopy (SEM) images were taken on a Hitachi High Technologies model SU8010 microscope. Analytical high-performance liquid chromatography (HPLC) experiments were performed on a JASCO model PU-4080i pump equipped with a JASCO model UV-4075 detector.

Materials. Unless otherwise noted, all reagents and solvents were purchased and used as received. Ethyl 3,5-bis((diethoxyphosphoryl)methyl)benzoate (**9**)[1], 3,4,5-tris(dodecyloxy)benzaldehyde (**10**)[2], (1*S*,2*S*)- and (1*R*,2*R*)-pseudonorephedrine (**6**^{S} and **6**^{R})[3], (1*R*,2*S*)-2-(methylamino)-1,2-diphenylethan-1-ol (**2**$_{(NMe)}^{S}$)[4], and (1*S*,2*R*)-2-methoxy-1,2-diphenylethan-1-amine (**2**$_{(OMe)}^{S}$)[5] were prepared according to the literature methods. Dodecane (Kanto), (1*R*,2*S*)- and (1*S*,2*R*)-2-amino-1,2-diphenylethanol (**2**^{S} and **2**^{R}; Wako), (1*S*,2*R*)- and (1*R*,2*S*)-1-amino-2-indanol (**3**^{S} and **3**^{R}; TCI), (1*S*,2*S*)- and (1*R*,2*R*)-1-amino-2-indanol (**4**^{S} and **4**^{R}; Sigma-Aldrich), (1*R*,2*S*)- and (1*S*,2*R*)-norephedrine (**5**^{S} and **5**^{R}; TCI), (*S*)- and (*R*)-phenylglycinol (**7**^{S} and **7**^{R}; Sigma-Aldrich), (*S*)- and (*R*)-1-amino-2-phenylethanol (**8**^{S} and **8**^{R}; Sigma-Aldrich) were purchased and used as received.

Preparation of the Dendritic Carboxylic Acid[6]

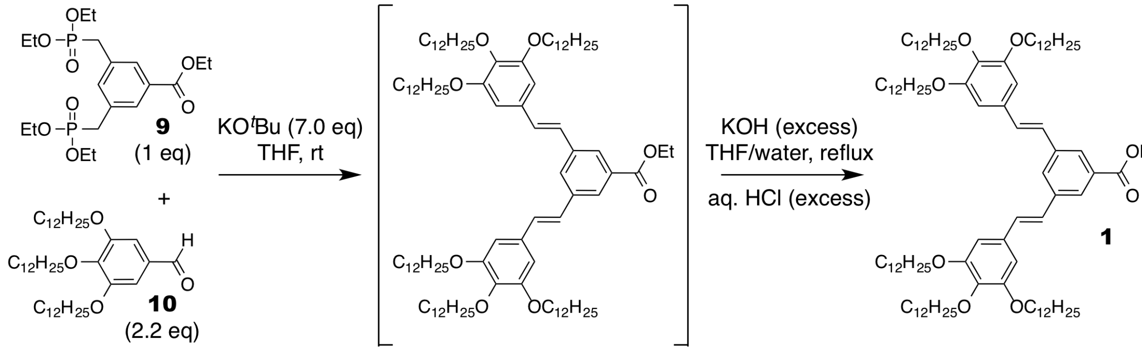

To a solution of ethyl 3,5-bis((diethoxyphosphoryl)methyl)benzoate (**9**; 1.20 g, 2.66 mmol) and 3,4,5-tris(dodecyloxy)benzaldehyde (**10**; 3.85 g, 5.84 mmol) in anhydrous THF (100 mL),

potassium *tert*-butoxide (2.09 g, 18.6 mmol) was portionwise added under Ar atmosphere at room temperature with magnetic stirring. After being stirred at room temperature for 48 h, the resulting mixture was treated with aqueous KOH (1.0 M, 55 mL) and heated under reflux with magnetic stirring for 96 h. The resulting mixture was then cooled to room temperature, acidified with aqueous HCl (1.0 M, 65 mL), and extracted with CH_2Cl_2 (2 × 100 mL). The combined organic layers were successively washed with water (50 mL) and brine (50 mL), dried over anhydrous Na_2SO_4, and concentrated to dryness. The resultant solid was subjected to column chromatography on silica gel eluted with hexane/EtOAc = 98/2–70/30, v/v, and then crystallized from EtOH to afford 3,5-bis((*E*)-3,4,5-tris(dodecyloxy)styryl)benzoic acid (**1**) as a pale yellow solid (1.60 g, 1.12 mmol, 42% yield). For ^{1}H and ^{13}C NMR spectra, see Supplementary Fig. 1.

FT-IR (KBr pellet): ν = 3695, 3162, 2922, 1698, 1583, 1506, 1469, 1428, 1388, 1335, 1318, 1237, 1119, 989, 953, 846, 806, 770, 720 cm^{-1}.

^{1}H NMR ($CDCl_3$/CD_3OD = 95:5, v/v, 600 MHz): δ = 8.09 (s, 2H, Ar), 7.75 (s, 1H, Ar), 7.11 (d, *J* = 16.2 Hz, 2H, C=CH), 7.00 (d, *J* = 16.2 Hz, 2H, C=CH), 6.73 (s, 4H, Ar), 4.02 (t, *J* = 6.0 Hz, 8H, OCH_2), 3.98 (t, *J* = 6.0 Hz, 4H, OCH_2), 1.83 (t, *J* = 6.9 Hz, 8H, OCH_2), 1.77 (t, *J* = 6.9 Hz, 4H, OCH_2), 1.5–1.2 (m, 108H, CH_2), 0.88 (t, 18H, *J* = 5.7 Hz, CH_3) ppm.

^{13}C NMR ($CDCl_3$/CD_3OD = 95:5, v/v, 150 MHz): 153.30, 138.50, 138.13, 132.15, 131.19, 130.25, 128.61, 126.53, 105.28, 73.62, 69.19, 31.94, 31.93, 30.34, 29.75, 29.72, 29.63, 29.46, 29.40, 29.37, 26.15, 22.69, 14.09 ppm.

MALDI-TOF-MS (dithranol): *m*/*z* calcd. for $C_{95}H_{162}O_8$ $[M]^+$ = 1431.23, found 1431.46.

Preparation of the Supramolecular Polymers in Dodecane

In a typical procedure, a $CHCl_3$ solution (800 μL) of the dendritic carboxylic acid **1** (4.0 mM, 3.2 μmol) and a $CHCl_3$ solution (800 μL) of the chiral amino alcohol $\mathbf{2}^{S}$ (4.0 mM, 3.2 μmol) were added to a glass vial, and the solvent was slowly evaporated to dryness under ambient conditions to afford the salt $\mathbf{1}\cdot\mathbf{2}^{S}$. The resultant salt was dissolved in dodecane (800 μL), heated to 348 K, and then cooled to the intended temperature at a rate of –0.5 K min^{-1} (for FT-IR spectroscopy) or –1.0 K min^{-1} (for UV/Vis and CD spectroscopies) to afford the dodecane solution of the supramolecular polymer of $\mathbf{1}\cdot\mathbf{2}^{S}$ (4.0 mM), which was immediately used for spectroscopic measurements.

Dodecane solutions of other supramolecular polymers were prepared in the same procedure, using the respective amino alcohols.

Fourier Transform Infrared (FT-IR) Spectroscopy

FT-IR spectra of the supramolecular polymers in dodecane were measured on a JASCO model FT/IR-4100 spectrometer equipped with a home-made temperature controller.
Resolution: 4.0 cm^{-1}. Range: 4000–600 cm^{-1}. Number of scans: 80.

A dodecane solution of the supramolecular polymer was held in an assembled cuvette with CaF_2 windows and cooled from 348 K to the intended temperature at a rate of 0.5 K min^{-1}. The FT-IR measurement was conducted just after the target temperature was reached.

UV/Vis and Circular Dichroism (CD) Spectroscopy

UV/Vis and CD spectra of the supramolecular polymers in dodecane were measured on a JASCO J-1500 spectropolarimeter equipped with a JASCO PTC-510 Peltier-type temperature controller.
Bandwidth: 2.0 nm. Response time: 2.0 s. Sensitivity: standard. Scan rate: 200 nm min^{-1}.

A dodecane solution of the supramolecular polymer was held in an assembled cuvette with quartz windows and a Teflon spacer with 0.1 mm path length. Only in the experiments of Supplementary Figs 6 and 22a, Teflon spacers with 0.5 mm and 0.2 mm path lengths were used, respectively.

Continuous monitoring of one sample at 354 nm reduced the CD intensity, most likely because of the photoisomerization and/or photodimerization of the stilbene units in **1** (Supplementary Fig. 4, ii). Such reduction of CD intensity was inevitable even when the intensity of incident light was lowered by reducing the bandwidth and response time to 0.5 nm and 0.5 s, respectively (Supplementary Fig. 4, iii). Therefore, the cooling curve was obtained by combining data measured by using freshly prepared samples for all of the temperatures from 348 to 283 K at 5-K step (Supplementary Fig. 4, i).

Synchrotron X-Ray Diffraction (XRD) Measurement

XRD measurement of the thermotropic liquid crystal of $\mathbf{1}\cdot\mathbf{2}^{S}$ was carried out at BL45XU in SPring-8 (Hyogo, Japan)[7]. Diffraction data were collected using Dectris model Pilatus 300K-W detectors. The incident X-ray beam (1.00 Å wavelength) was monochromated by a diamond (1 1 1) double-crystal monochromator. The sample-to-detector distance was 260 mm. Scattering vector q and position of an incident X-ray beam on the detector were calibrated using several orders of layer reflections from silver behenate. The sample of $\mathbf{1}\cdot\mathbf{2}^{S}$ was placed into a 1.5 mm-ϕ glass capillary, heated to isotropic melt at 150 °C, cooled to 120 °C at a rate of –1.0 °C min^{-1} and exposed at 120 °C to an X-ray beam for 1.0 s.

Atomic-Force Microscopy (AFM) Measurement

AFM images of the supramolecular polymers were taken on an Oxford Instruments model Cypher AFM system, using Olympus OMCL-AC160TS sharpened tetrahedral-shaped silicon cantilevers (resonant frequency = 300 kHz; spring constant = 26 N m^{-1}) in a tapping mode. Samples were prepared by spin-coating the dodecane solutions of supramolecular polymers on highly oriented pyrolytic graphite (HOPG) substrates.

Fast Fourier Translation (FFT) Analysis of AFM Images[8]

A square-shaped region (38 nm × 38 nm) was trimmed from the AFM image of the supramolecular polymer in Fig. 4, where the trimming angle was adjusted so that the direction of the fibres of the supramolecular polymer aligned in the vertical direction. The square-shaped image thus trimmed was converted into a grey scale image and then subjected to FFT, using software ImageJ[9]. When spots appeared at the second and forth quadrants of the FFT image, the helical handedness of the supramolecular polymer was determined to be left-handed. When spots appeared at the first and third quadrants of the FFT image, the helical handedness of the supramolecular polymer was determined to be right-handed. For details, see also Supplementary Fig. 12.

Enantio-Separation of the Amino Alcohols by the Supramolecular Polymer

Protocol 1. Separately prepared dodecane solutions (4.0 mM) of **1·3*S*** (210 µL), **1·3*R*** (210 µL), and **1·2*S*** (420 µL) were sequentially added to a glass vial at 298 K and left to stand at 298 K for 2 h, where a precipitate formed. The resultant suspension was subjected to centrifugation, and the supernatant was extracted with 1,000 µL of $HClO_4$ (pH 2.0)/MeOH = 80/20, v/v. The aqueous layer was directly injected to chiral HPLC (see below) to determine the enantiomeric composition and recovery yield of the mixture of **3*S*** and **3*R*** from the HPLC peak areas. Chiral separation of **4*S*** and **4*R*** was conducted in the same procedure.

Protocol 2. A $CHCl_3$ solution (218 µL) of **3*S*** (4.0 mM, 0.87 µmol) and a $CHCl_3$ solution (218 µL) of **3*R*** (4.0 mM, 0.87 µmol) was added to a glass vial, and the solvent was slowly evaporated to dryness under ambient conditions. To the glass vial containing solid **3*S***/**3*R***, a dodecane solution (1,200 µL) containing **1·2*S*** (3.0 mM, 3.60 µmol) and free acid **1** (1.0 mM, 1.20 µmol) was added at 298 K. The mixture was left to stand at 298 K for 1.3 h, where the mixture once became homogeneous, and then precipitate formed. The resultant suspension was subjected to centrifugation, and the supernatant was extracted with 4,000 µL of $HClO_4$ (pH 2.0)/MeOH = 80/20,

v/v. The aqueous layer was directly injected to chiral HPLC (see below) to determine the enantiomeric composition and recovery yield of the mixture of $\mathbf{3}^{S}$ and $\mathbf{3}^{R}$ from the HPLC peak areas.

Protocol 3. A $CHCl_3$ solution (200 μL) of $\mathbf{3}^{S}$ (4.0 mM, 0.80 μmol) and a $CHCl_3$ solution (200 μL) of $\mathbf{3}^{R}$ (4.0 mM, 0.80 μmol) was added to a glass vial, and the solvent was slowly evaporated to dryness under ambient conditions. To the glass vial containing solid $\mathbf{3}^{S}$/$\mathbf{3}^{R}$, a dodecane solution (400 μL) containing $\mathbf{1}\cdot\mathbf{2}^{S}$ (4.0 mM, 1.60 μmol) was added at 298 K. The mixture was left to stand at 298 K for 1.3 h, where the mixture once became homogeneous, and then precipitate formed. The resultant suspension was subjected to centrifugation, and the supernatant was extracted with 4,000 μL of $HClO_4$ (pH 2.0)/MeOH = 80/20, v/v. The aqueous layer was directly injected to chiral HPLC (see below) to determine the enantiomeric composition and recovery yield of the mixture of $\mathbf{3}^{S}$ and $\mathbf{3}^{R}$ from the HPLC peak areas. Enantio-separation of $\mathbf{4}^{S}$ and $\mathbf{4}^{R}$ was conducted in the same procedure.

Chiral HPLC. Chiral HPLC analysis of the mixture of $\mathbf{3}^{S}$ and $\mathbf{3}^{R}$ was performed on a JASCO model PU-4080i pump equipped with a model UV-4075 detector. Column: Daicel CHIRALCEL CROWNPAK CR(+) (4.0 mm × 150 mm). Eluent: pH 2.0 aqueous $HClO_4$/MeCN = 95/5, v/v. Temperature: 273 K. Flow rate: 0.60 mL min^{-1}. Injection volume: 5.0 μL. Detection: UV absorption at 210 nm. Elution time: 7.5 min ($\mathbf{3}^{R}$) and 9.1 min ($\mathbf{3}^{S}$). Chiral HPLC analysis of the mixture of $\mathbf{4}^{S}$ and $\mathbf{4}^{R}$ was performed under the same conditions as those of the case of $\mathbf{3}^{S}$ and $\mathbf{3}^{R}$. Elution time: 7.5 min ($\mathbf{4}^{R}$) and 9.7 min ($\mathbf{4}^{S}$).

Reuse of the Carboxylic Acid. The first run of the enantio-separation was conducted following protocol 1 as described above. Thus, separately prepared dodecane solutions (4.0 mM) of $\mathbf{1}\cdot\mathbf{3}^{S}$ (210 μL), $\mathbf{1}\cdot\mathbf{3}^{R}$ (210 μL), and $\mathbf{1}\cdot\mathbf{2}^{S}$ (420 μL) were mixed and left to stand at 298 K for 2 h, where a precipitate formed. The supernatant and precipitate were separated by centrifugation. The supernatant was washed with 2,000 μL of $HClO_4$ (pH 2.0)/MeOH = 80/20, v/v, while the precipitate was suspended in 600 μL of dodecane and washed with 2,000 μL of $HClO_4$ (pH 2.0)/MeOH = 80/20, v/v. The dodecane layers were combined and dried under reduced pressure (~1 mmHg) at 298 K for 24 h to afford a dodecane suspension of **1** (~3.2 μmol). The suspension was mixed with a $CHCl_3$ solution of $\mathbf{2}^{S}$ (4.0 mM, 800 μL), concentrated under reduced pressure (~1 mmHg) at 298 K for 48 h, and diluted with dodecane to adjust the total volume to 800 μL to afford a dodecane solution of $\mathbf{1}\cdot\mathbf{2}^{S}$ (4.0 mM), which was used for the second run of the enantio-separation.

Supplementary Figures (Supplementary Figs 1–32)

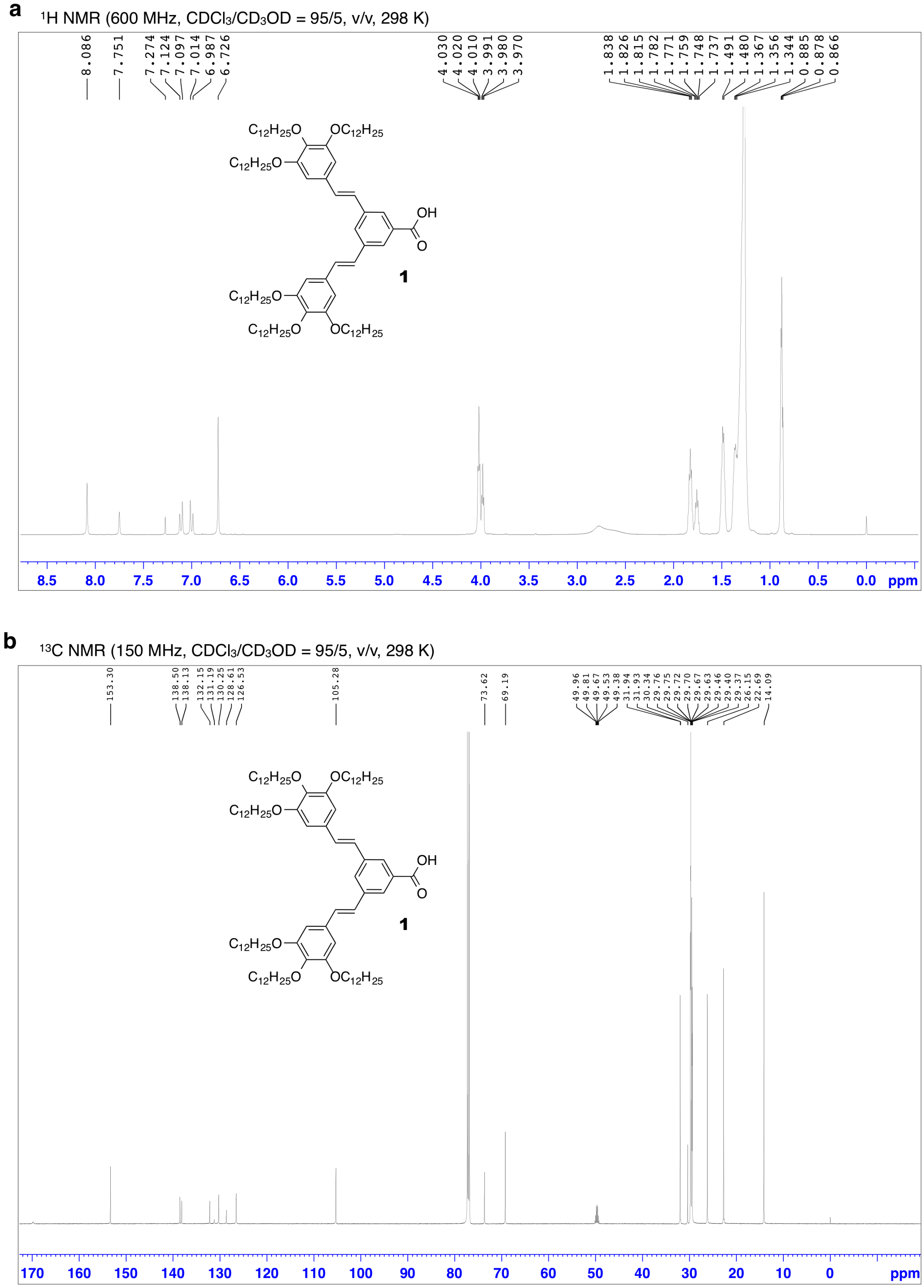

Supplementary Fig. 1 | Characterization of the dendritic carboxylic acid 1.

a,**b**, ^{1}H (600 MHz) and ^{13}C (150 MHz) NMR spectra of **1** in $CDCl_3/CD_3OD$ = 95/5, v/v, at 298 K.

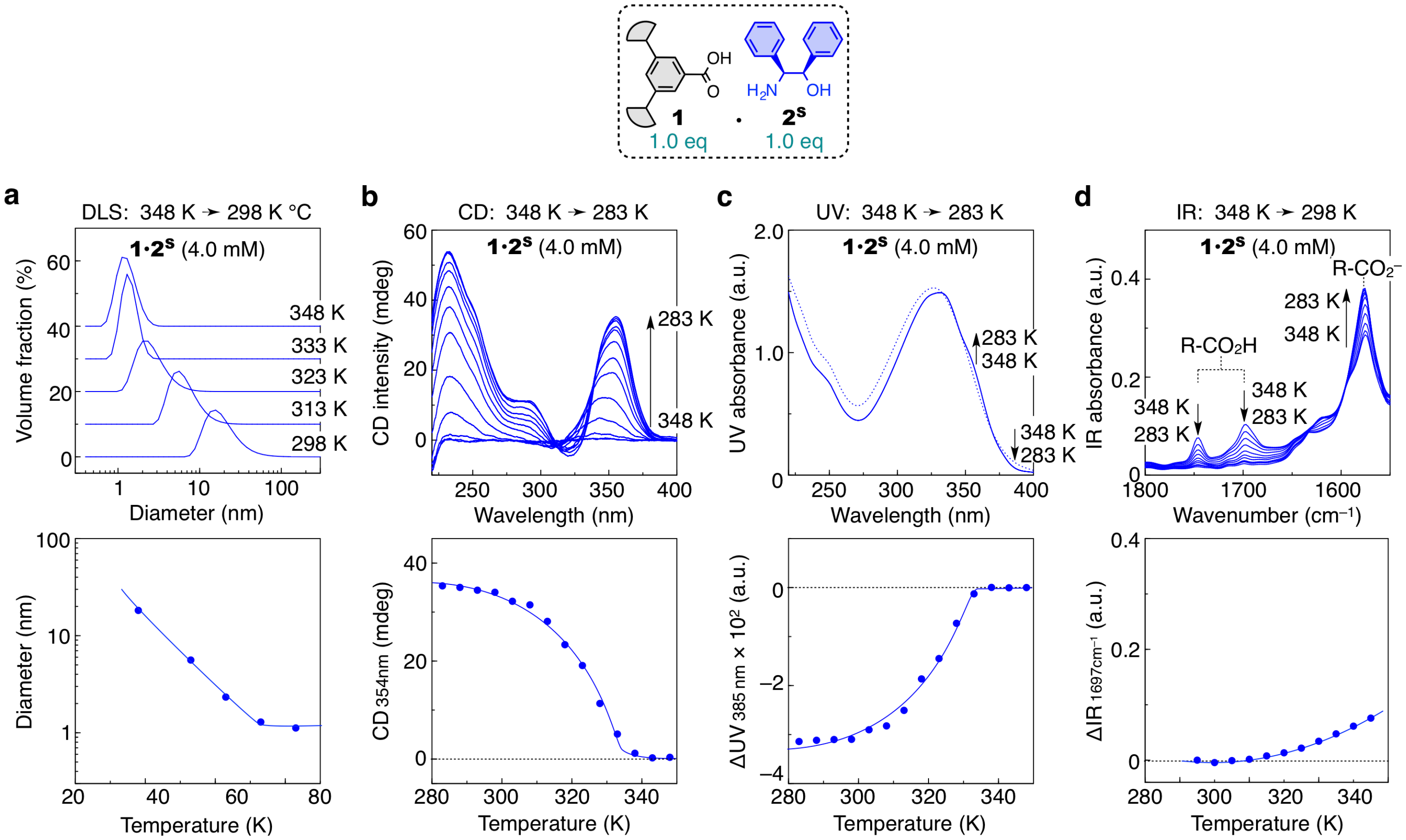

Supplementary Fig. 2 | Temperature-dependent changes of 1·2^{S} in dodecane.

a–**d**, Variable-temperature spectroscopic profiles of **1·2^{S}** in dodecane (4.0 mM).

Upper: DLS (**a**), CD (**b**), UV (**c**), and IR (**d**) spectra.

Lower: Plots of DLS hydrodynamic diameter (**a**), CD intensity at 354 nm (**b**), changes in UV absorption at 385 nm (**c**), and changes in IR absorption at 1697 cm^{-1} (**d**) against temperature.

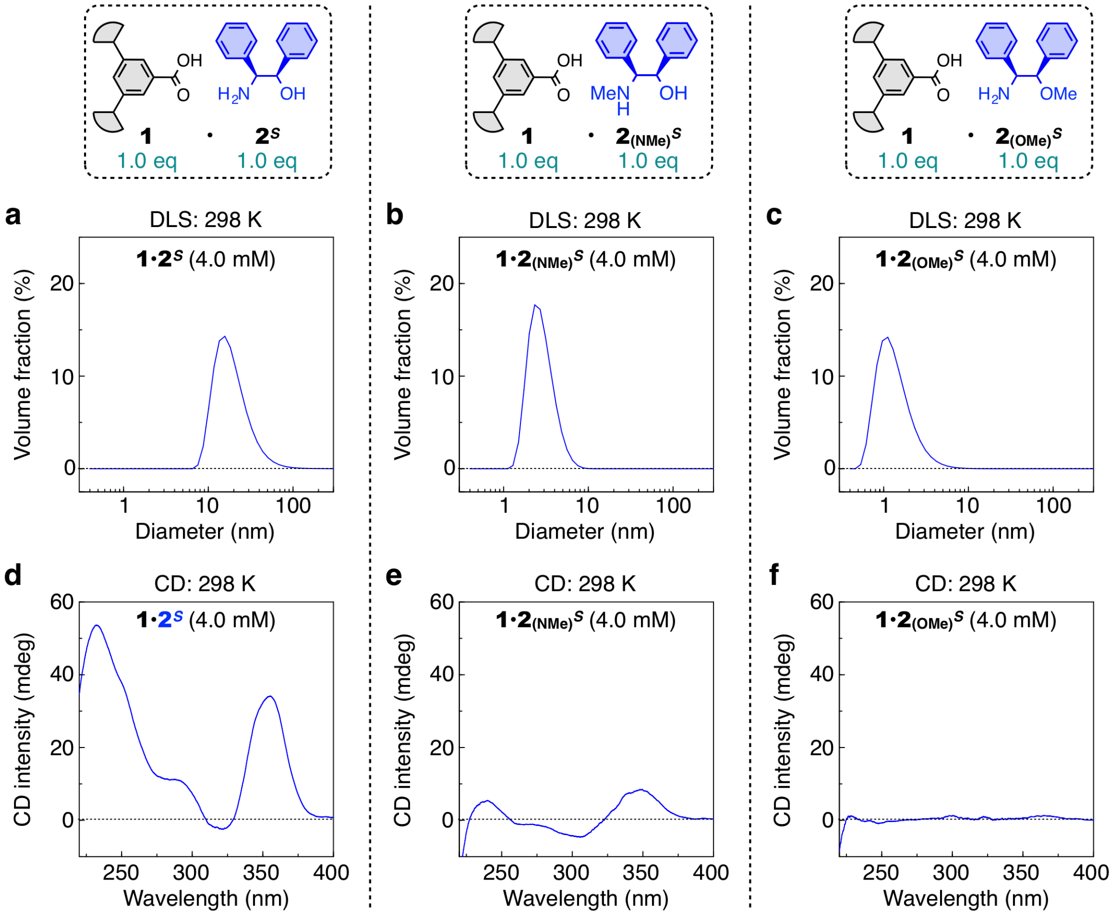

Supplementary Fig. 3 | Role of hydrogen-bonding sites on the formation of helical supramolecular polymer.

a–**c**, DLS traces of **1**•**2**^{S} (**a**), **1**•**2**$_{\mathbf{(NMe)}}$^{S} (**b**), and **1**•**2**$_{\mathbf{(OMe)}}$^{S} (**c**) in dodecane (4.0 mM) at 298 K.

d–**f**, CD spectra of **1**•**2**^{S} (**d**), **1**•**2**$_{\mathbf{(NMe)}}$^{S} (**e**), and **1**•**2**$_{\mathbf{(OMe)}}$^{S} (**f**) in dodecane (4.0 mM) at 298 K.

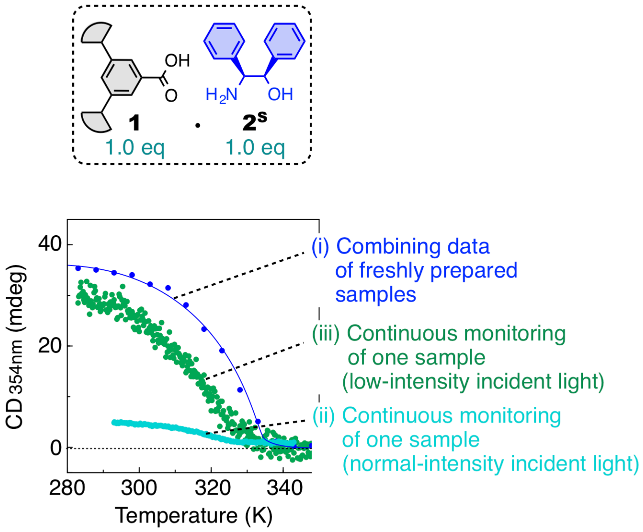

Supplementary Fig. 4 | Photodegradation of 1 during continuous CD-monitoring at 354 nm.

Cooling curves from 348 to 283 K at –1.0 K min^{-1} of **1**•**2**^{S} in dodecane (4.0 mM) monitored with CD intensity at 354 nm. For details see Supplementary Methods.

(i) Cooling curve obtained by combining data measured by using freshly prepared samples for all of the temperatures at every 5 K step.

(ii) Cooling curve obtained by continuously monitoring of one sample.

(iii) Cooling curve obtained by continuously monitoring of one sample using low-intensity incident light.

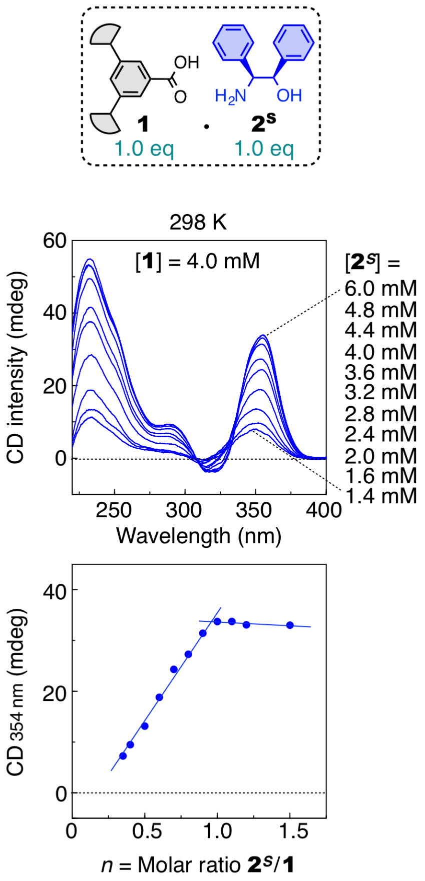

Supplementary Fig. 5 | Titration experiment of 1 with 2^S in dodecane.

Upper: CD spectra of the mixtures of **1** (4.0 mM) with various amounts **2^S** (1.4–6.0 mM) in dodecane at 298 K.

Lower: Plot of CD intensity at 354 nm against the molar ratio **2^S/1**.

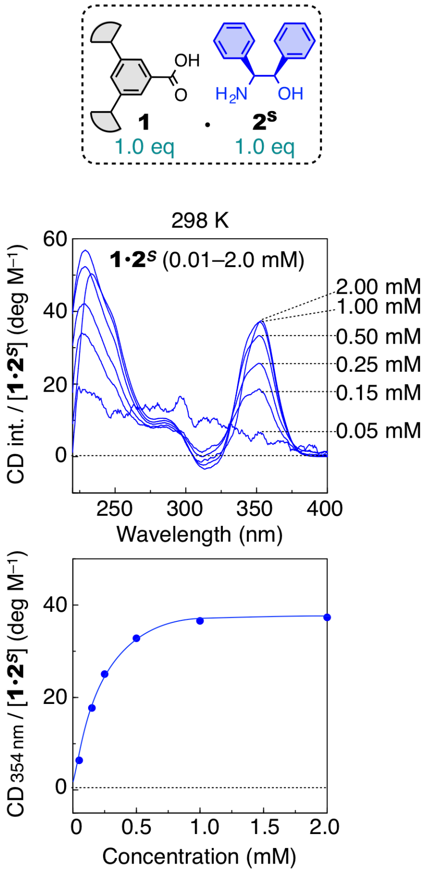

Supplementary Fig. 6 | Dilution experiment of 1•2^S in dodecane.

Upper: Concentration-normalized CD spectra of **1•2^S** in dodecane at 298 K with various concentrations (0.05–2.0 mM).

Lower: Plot of concentration-normalized CD intensity at 354 nm against concentration of **1•2^S**.

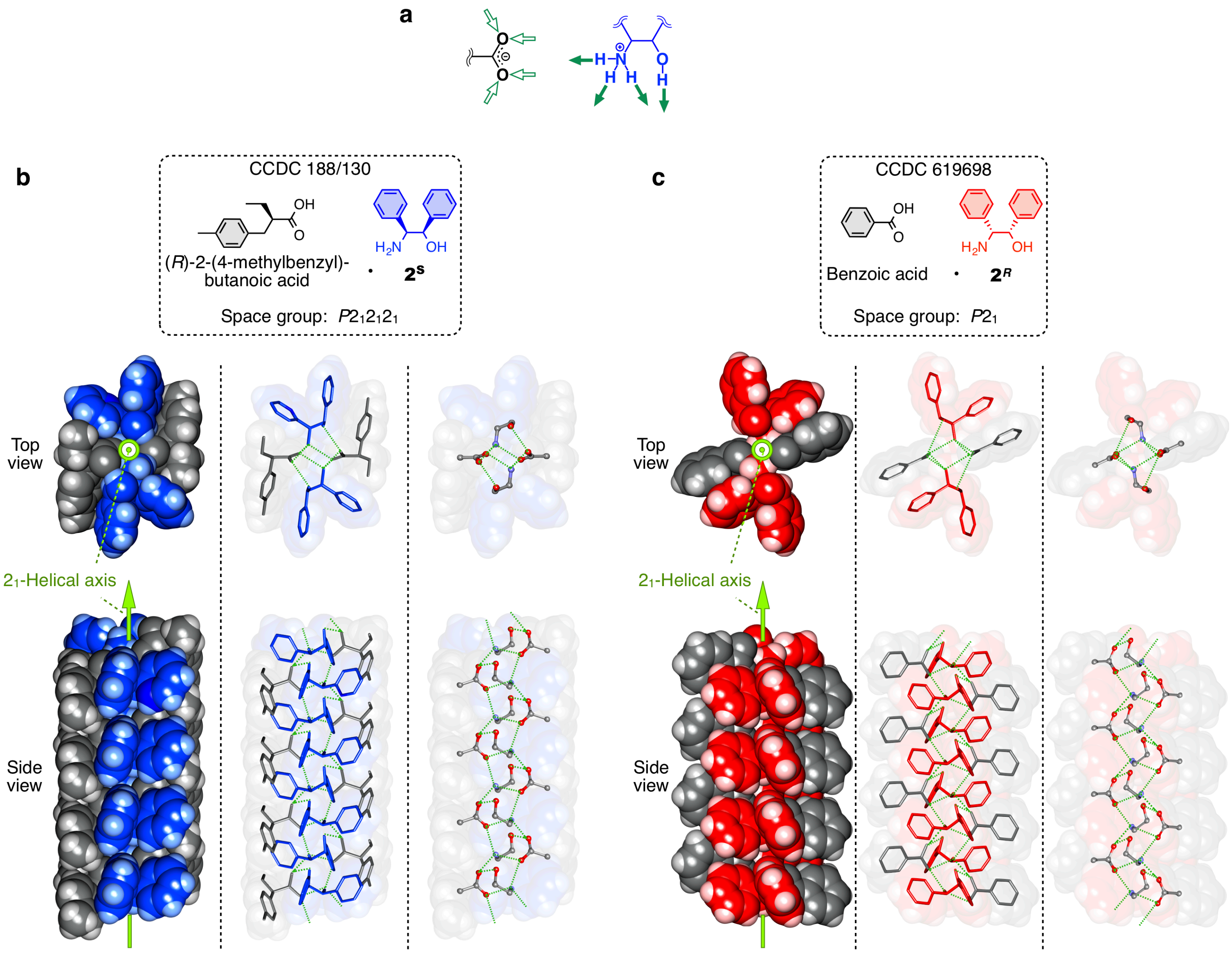

Supplementary Fig. 7 | X-ray crystal structures of the model salts without long alkyl chains.

a, Hydrogen-donating sites in a carboxylic acid (R-CO_2^- form; left) and hydrogen-accepting sites in an amino alcohol (R'-NH_3^+ form; right).

b,**c**, X-ray crystal structures of a carboxylate salt of **2*S*** (**b**; CCDC188/130)[10] and a carboxylate salt of **2*R*** (**c**; CCDC 619698)[11] viewed from the top (upper) and side (lower) of their hydrogen-bonded columnar network (green doted lines) with CPK (left), stick (middle), and ball-and-stick (right) representations. These structures adopt the space group of $P2_12_12_1$ and $P2_1$, respectively, and have 2_1-helical axes as shown in the left panels. In the left and middle panels, molecules of **1**, **2*S***, and **2*R*** are shown in grey, blue, and red, respectively. In the middle and right panels, hydrogen atoms are omitted. In the right panels, only the parts of molecules involved in the hydrogen-bonded networks are shown, where carbon, oxygen, and nitrogen atoms are shown in grey, red, and blue, respectively.

a

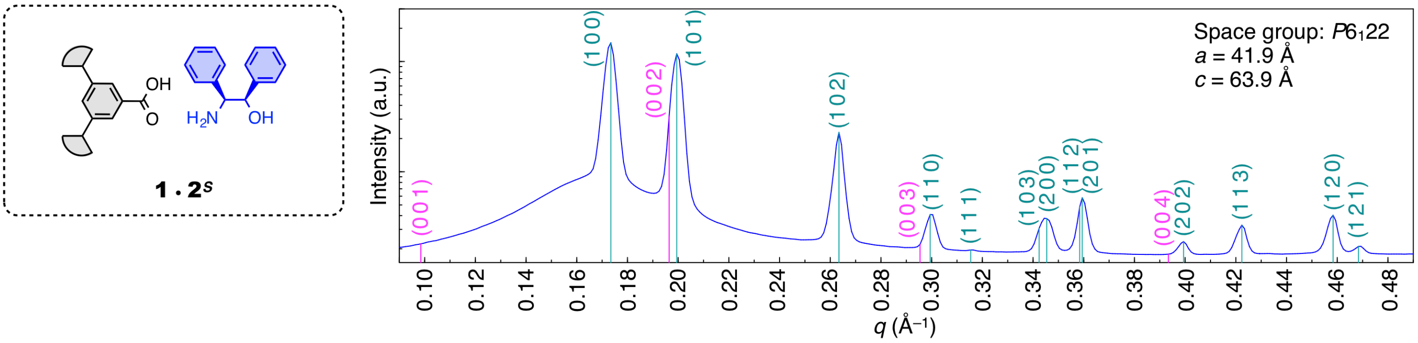

b

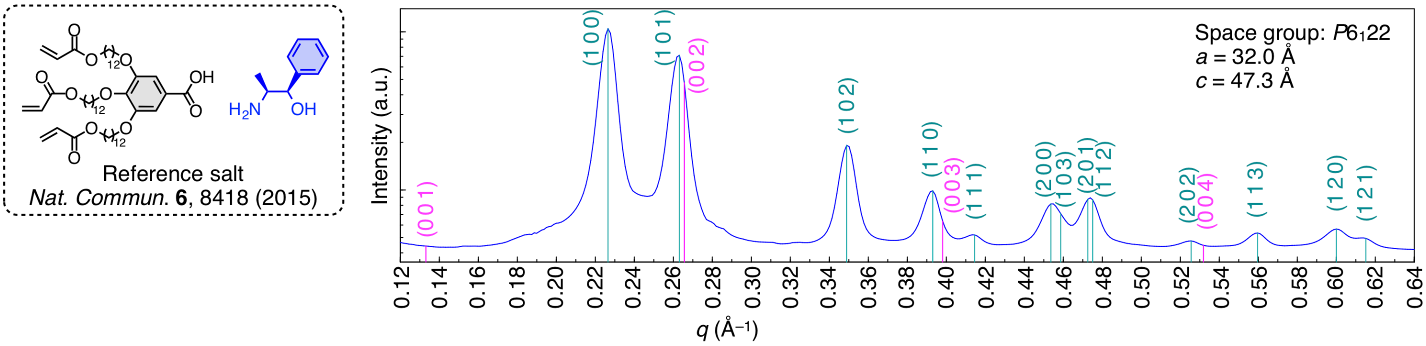

c

Miller index	Extinction rule	**1·2**^{S}		Reference salt	
(h k l)	for $P6_122$	$q_{calcd.}$ (Å^{-1})[a]	$q_{obsd.}$ (Å^{-1})	$q_{calcd.}$ (Å^{-1})[a]	$q_{obsd.}$ (Å^{-1})
(0 0 1)	Disappear[c]	0.098	n.d.[d]	0.133	n.d.[d]
(1 0 0)	Appear	0.173	0.173	0.227	0.227
(0 0 2)	Disappear[c]	0.197	n.d.[d]	0.266	n.d.[d]
(1 0 1)	Appear	0.199	0.199	0.263	0.263
(1 0 2)	Appear	0.262	0.263	0.349	0.349
(0 0 3)	Disappear[c]	0.295	n.d.[d]	0.398	n.d.[d]
(1 1 0)	Appear	0.300	0.299	0.393	0.393
(1 1 1)	Appear	0.315	0.315	0.415	0.415
(1 0 3)	Appear	0.342	0.344[e]	0.459	0.454[e]
(2 0 0)	Appear	0.346	0.344[e]	0.454	0.454[e]
(1 1 2)	Appear	0.358	0.359[e]	0.474	0.474[e]
(2 0 1)	Appear	0.360	0.359[e]	0.473	0.474[e]
(0 0 4)	Disappear[c]	0.393	n.d.[d]	0.531	n.d.[d]
(2 0 2)	Appear	0.398	0.399	0.526	0.523
(1 1 3)	Appear	0.420	0.422	0.560	0.560
(1 2 0)	Appear	0.458	0.458	0.600	0.600
(1 2 1)	Appear	0.468	0.468	0.615	0.614

[a] q value calculated for a hexagonal lattice with the lattice constants of a = 41.9 Å and c = 63.9 Å.
[b] q value calculated for a hexagonal lattice with the lattice constants of a = 32.0. Å and c = 47.3 Å.
[c] In the space group of $P6_122$, the reflections of (0 0 l) should disappear except for l = 6n.
[d] Reflection was not detected.
[e] Two reflections were overlapped.

Supplementary Fig. 8 | XRD profiles of 1·2^S and a reference salt in liquid crystalline states.

a,**b**, XRD profiles of **1·2**^{S} (**a**) and the reference salt (**b**)[12]. For each Miller index, the *q* value of the reflection is calculated and shown in a solid line, with supposing the lattice constants in the right top of the graph. Reflections that should appear and disappear due to the extinction rule for $P6_122$ are shown in green and magenta, respectively. The *q*-ranges of the graphs are adjusted so that the reflections with the same Miller index in **a** and **b** appear at similar regions.

c, Comparison of the calculated and observed *q* values of the reflections in **a** and **b**.

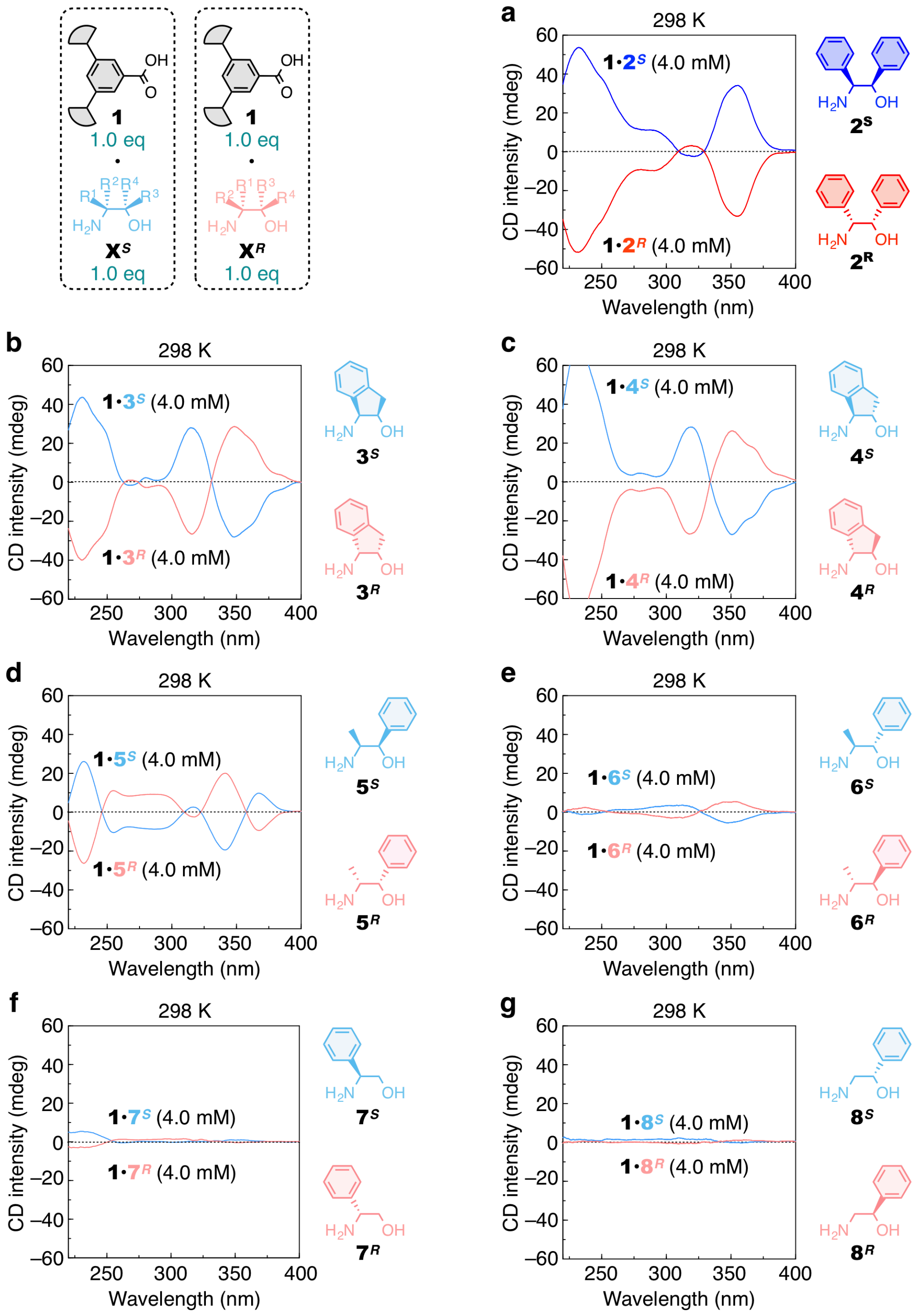

Supplementary Fig. 9 | Supramolecular polymers with modular chiral auxiliaries.

a–g, CD spectra of various salts (4.0 mM) in dodecane at 298 K: **1•2^S/1•2^R** (**a**), **1•3^S/1•3^R** (**b**), **1•4^S/1•4^R** (**c**), **1•5^S/1•5^R** (**d**), **1•6^S/1•6^R** (**e**), **1•7^S /1•7^R** (**f**) and **1•8^S/1•8^R** (**g**).

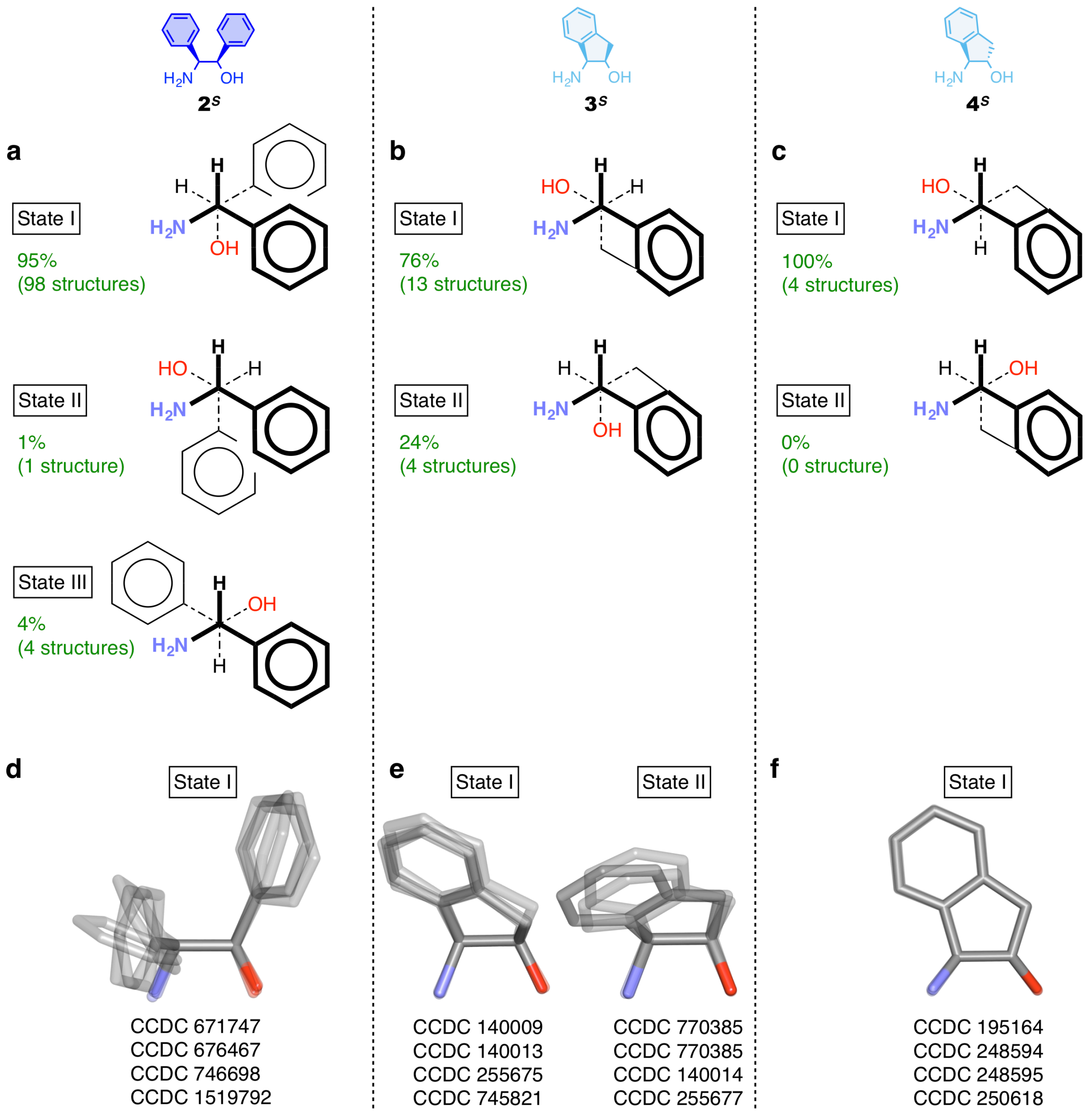

Supplementary Fig. 10 | Cambridge Structural Database (CSD) survey for the conformational stability for the amino alcohols.

a–**c**, Possible conformational states of **2**^{S} (**a**), **3**^{S} (**b**) and **4**^{S} (**c**) viewed along the C^1–C^2 bonds. All crystal structures deposited in CSD of the salts of **2**^{S}, **3**^{S} and **4**^{S} and their derivatives including the antipodes deposited were retrieved and checked in terms of the conformational states. For each conformational state, the number and probability of crystal structures in which the amino alcohol adopts the corresponding conformation is shown in green.

d–**f**, Superimposition of structures of the conformers of **2**^{S} and its derivatives (**d**), **3**^{S} and its derivatives (**e**) and **4**^{S} and its derivatives (**f**). Four representative structures were shown for each conformational state. The structures of the antipodes were inverted. Hydrogen atoms and substituent(s) on the aromatic ring are omitted.

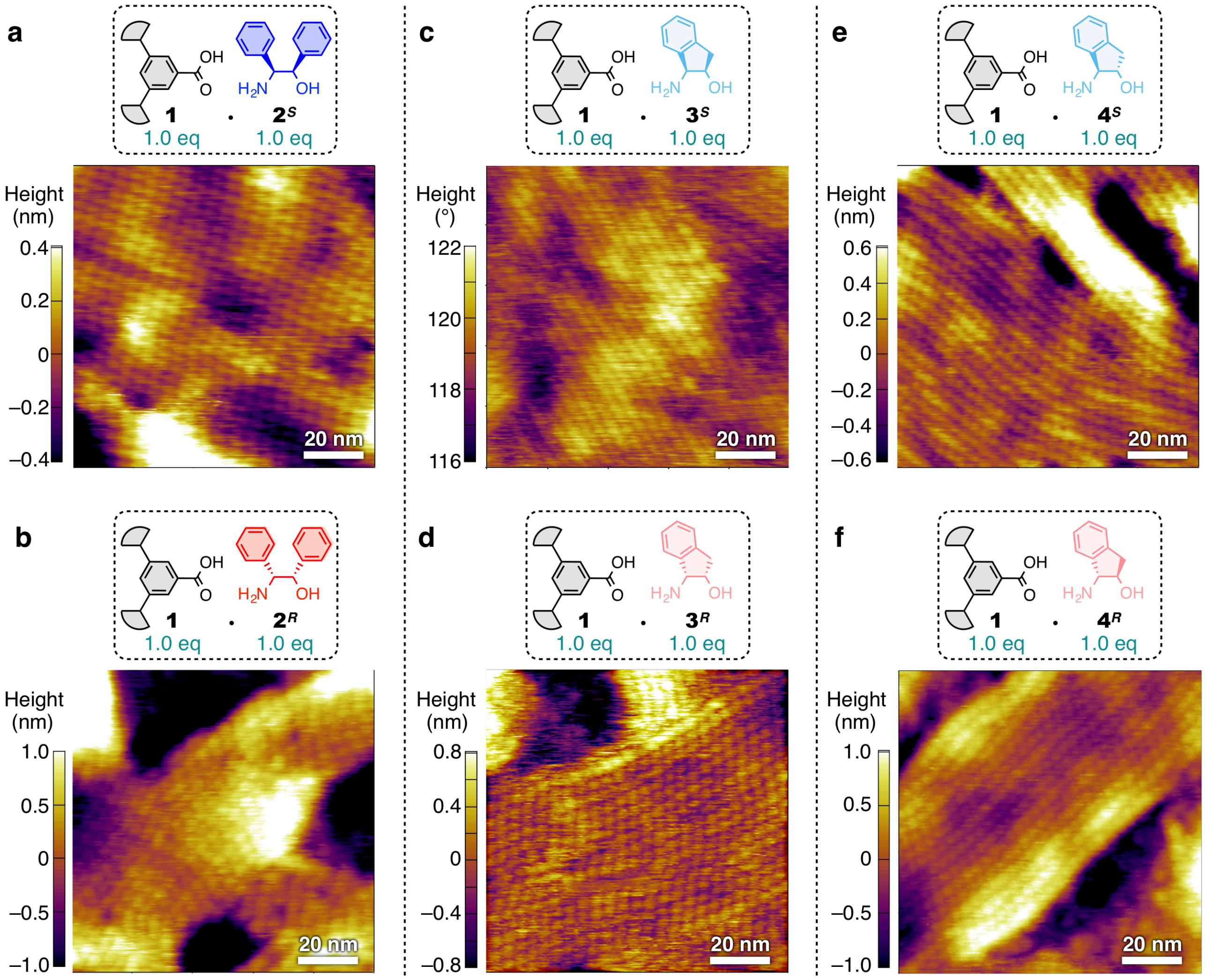

Supplementary Fig. 11 | Visualization of the helical supramolecular polymers by AFM.

a–f, AFM height images of the salts spin-coated on HOPG substrates from dodecane solutions (4.0 mM): **1•2**^{S} (**a**), **1•2**^{R} (**b**), **1•3**^{S} (**c**), **1•3**^{R} (**d**), **1•4**^{S} (**e**) and **1•4**^{R} (**f**).

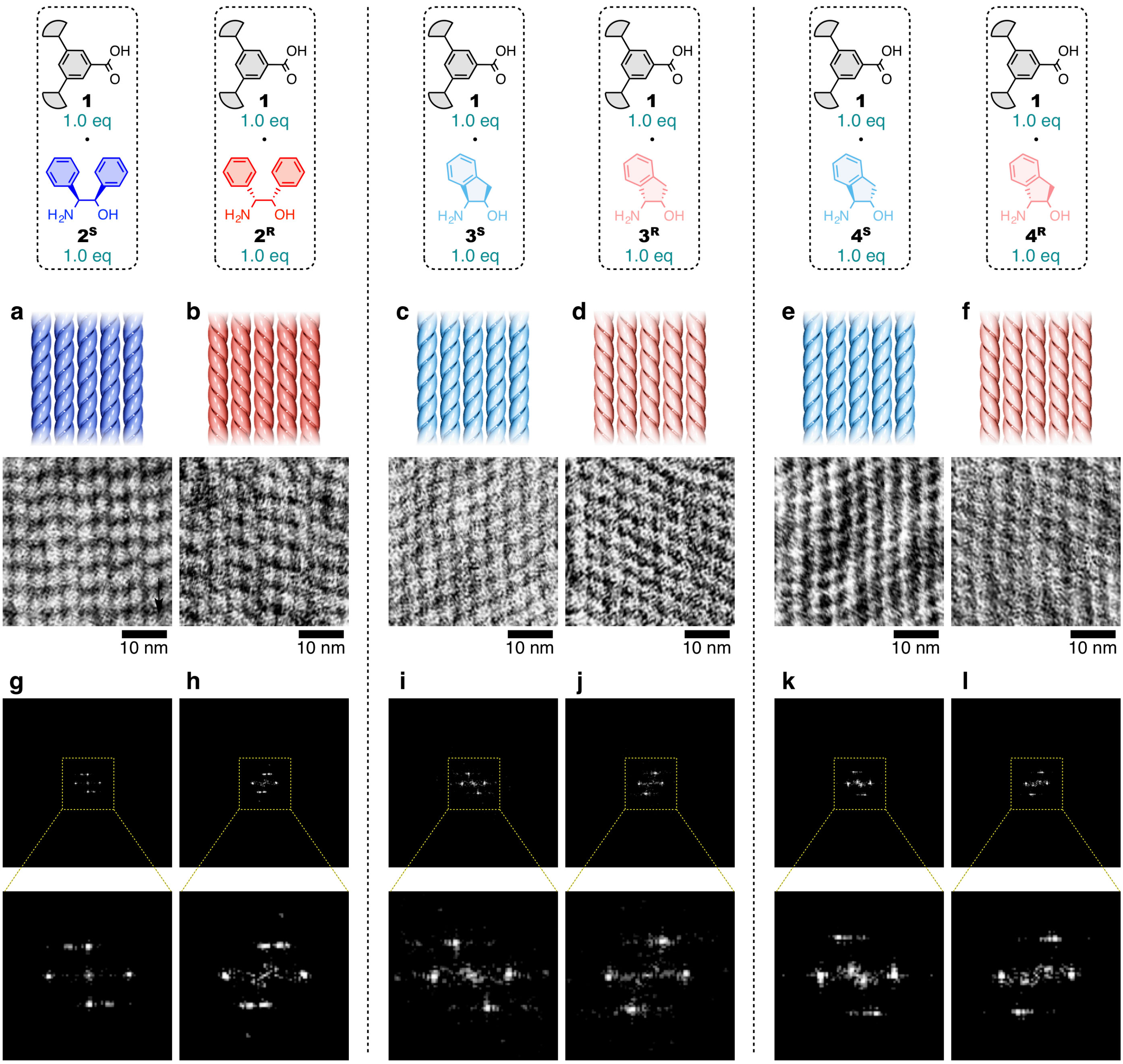

Supplementary Fig. 12 | Determination of the helical handedness of the supramolecular polymers by AFM[8]**.**

a–f, AFM images subjected to FFT in **g–l**: **1•2**^{S} (**a**), **1•2**^{R} (**b**), **1•3**^{S} (**c**), **1•3**^{R} (**d**), **1•4**^{S} (**e**) and **1•4**^{R} (**f**). From the AFM phase/amplitude images in Fig. 4, square-shaped regions (38 nm × 38 nm) containing vertically oriented polymer fibres were trimmed and converted into grey-scale images.

g–l, FFT images obtained from the AFM images in **a–f**: **1•2**^{S} (**g**), **1•2**^{R} (**h**), **1•3**^{S} (**i**), **1•3**^{R} (**j**), **1•4**^{S} (**k**) and **1•4**^{R} (**l**). For details, see Supplementary Methods.

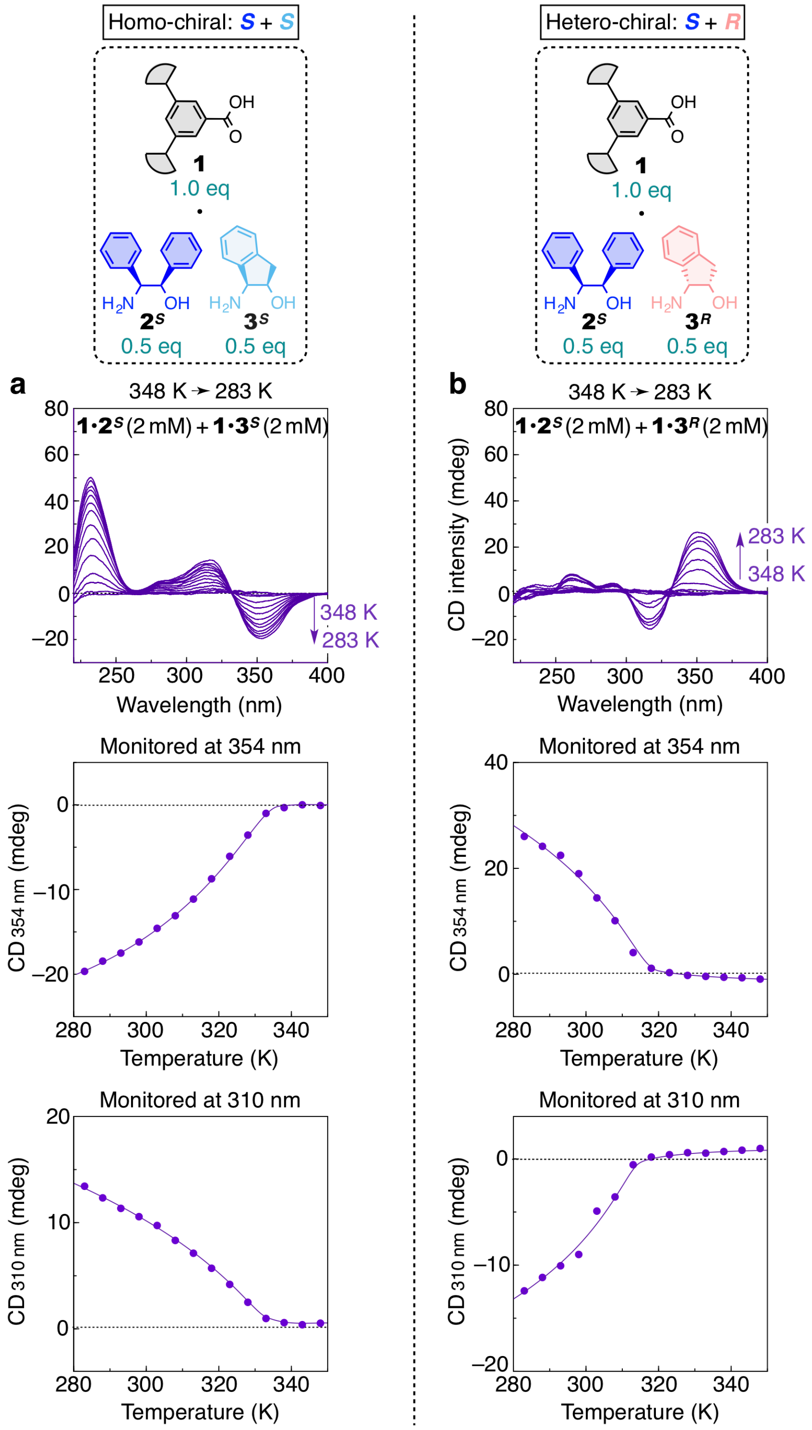

Supplementary Fig. 13 | Supramolecular copolymerization of two monomers upon cooling.

a,**b**, Variable-temperature CD profiles at every 5 K step of **1•2**^{S} and **1•3**^{S} in dodecane ([**1•2**^{S}] = [**1•3**^{S}] = 2.0 mM; **a**) and **1•2**^{S} and **1•3**^{R} in dodecane ([**1•2**^{S}] = [**1•3**^{R}] = 2.0 mM; **b**) measured upon cooling from 348 to 283 K at –1.0 K min^{-1}.

Upper: CD spectra.

Middle and lower: Cooling curves monitored with CD intensity at 354 nm (middle) and 310 nm (lower).

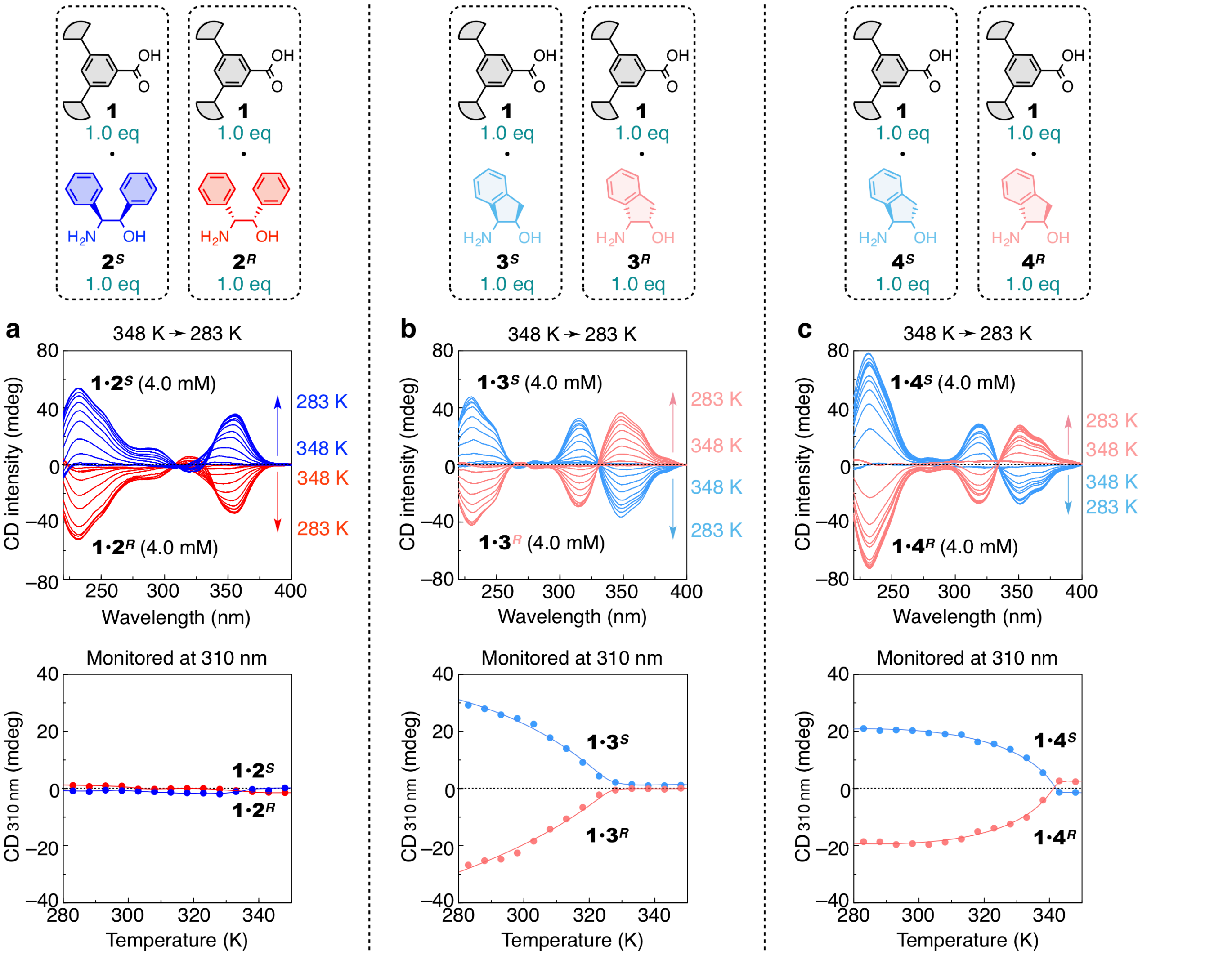

Supplementary Fig. 14 | Cooling curves of the helical supramolecular polymers monitored with CD intensity at 310 nm.

a–c, Variable-temperature CD profiles at every 5 K step of **1•2**^{S}/**1•2**^{R} (**a**), **1•3**^{S}/**1•3**^{R} (**b**) and **1•4**^{S}/**1•4**^{R} (**c**) in dodecane (4.0 mM) measured upon cooling from 348 to 283 K at –1.0 K min^{-1}.

Upper: CD spectra (same as Fig. 3a–c, upper).

Lower: Cooling curves monitored with CD intensity at 310 nm.

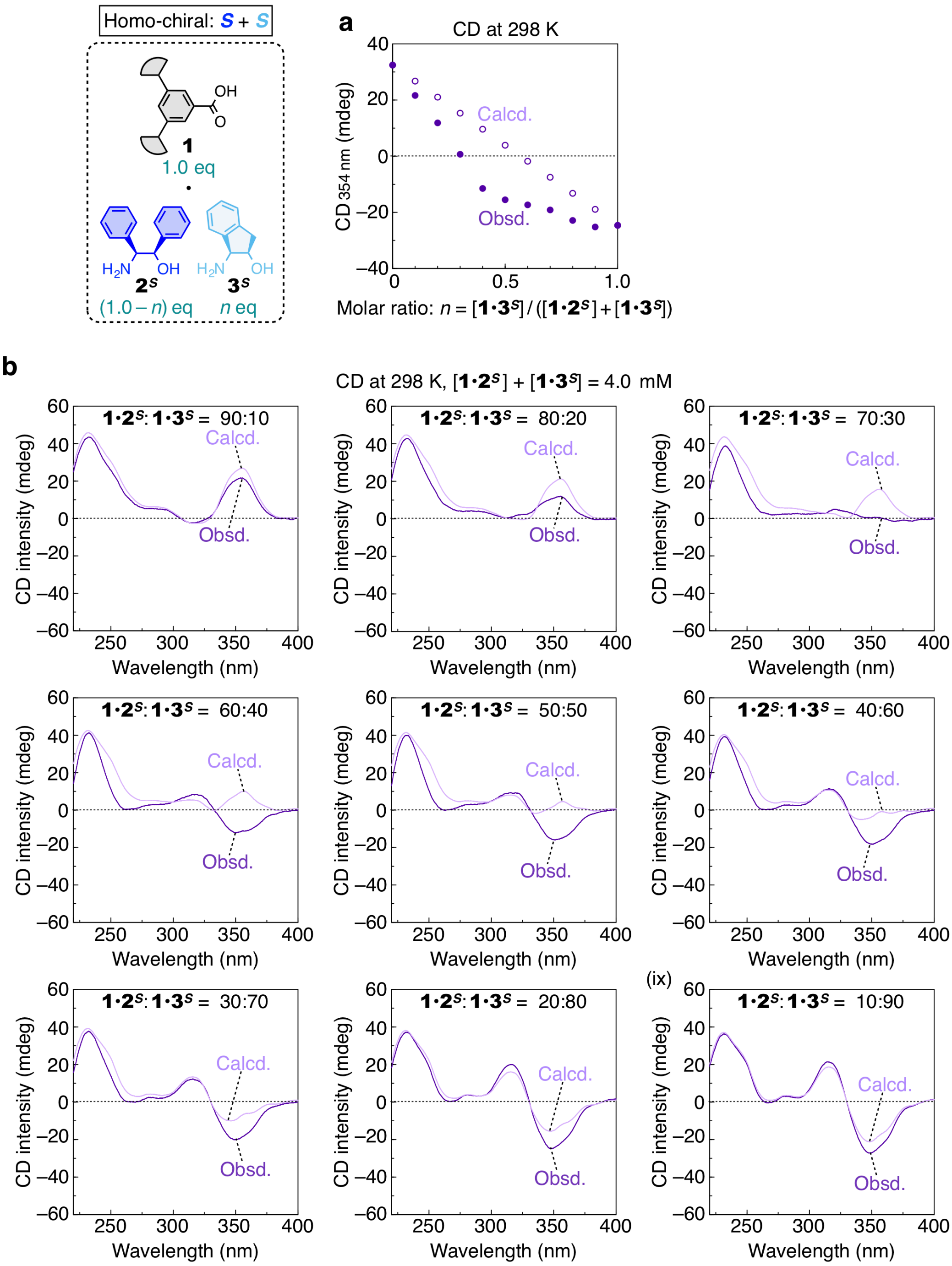

Supplementary Fig. 15 | CD profiles of the mixtures of homo-chiral monomers with various mixing ratios.

a,**b**, Obsd.: CD profiles measured for the mixtures of **1•2**^{S} and **1•3**^{S} in dodecane ([**1•2**^{S}] + [**1•3**^{S}] = 4.0 mM) at 298 K with various molar ratios (**1•2**^{S}:**1•3**^{S} = 90:10–10:90).

Calcd.: CD profiles calculated as linear sums of the CD profiles of **1•2**^{S} in dodecane (4.0 mM) and those of **1•3**^{S} in dodecane (4.0 mM) separately measured at 298 K.

Plots of CD intensity at 354 nm against the molar ratio of **1•2**^{S} and **1•3**^{S} (**a**) and CD spectra (**b**).

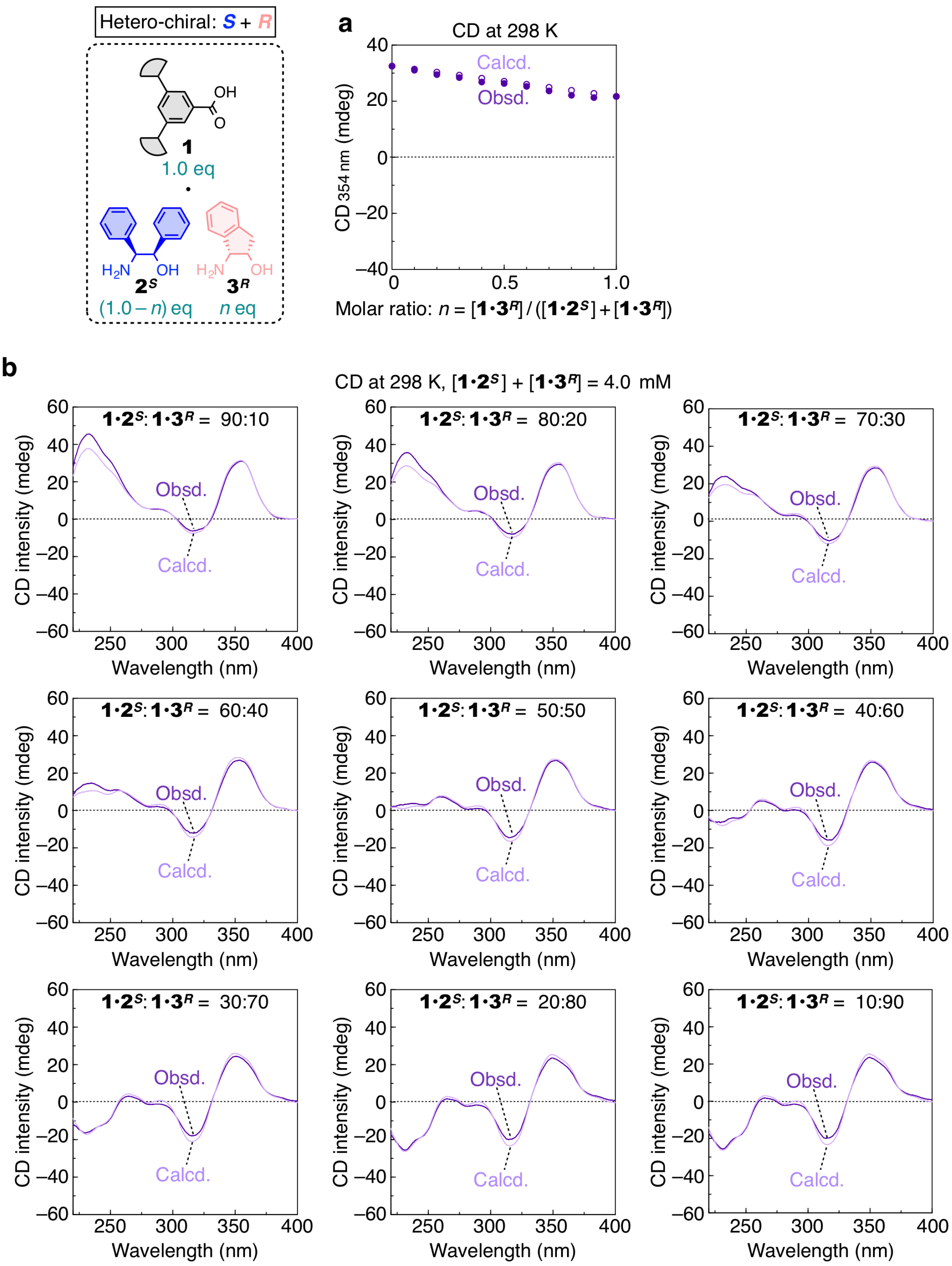

Supplementary Fig. 16 | CD profiles of the mixtures of hetero-chiral monomers with various mixing ratios.

a,**b**, Obsd.: CD profiles measured for the mixtures of **1•2**^{S} and **1•3**^{R} in dodecane ([**1•2**^{S}] + [**1•3**^{R}] = 4.0 mM) at 298 K with various molar ratios (**1•2**^{S}:**1•3**^{R} = 90:10–10:90).

Calcd.: CD profiles calculated as linear sums of the CD profiles of **1•2**^{S} in dodecane (4.0 mM) and those of **1•3**^{R} in dodecane (4.0 mM) separately measured at 298 K.

Plots of CD intensity at 354 nm against the molar ratio of **1•2**^{S} and **1•3**^{R} (**a**) and CD spectra (**b**).

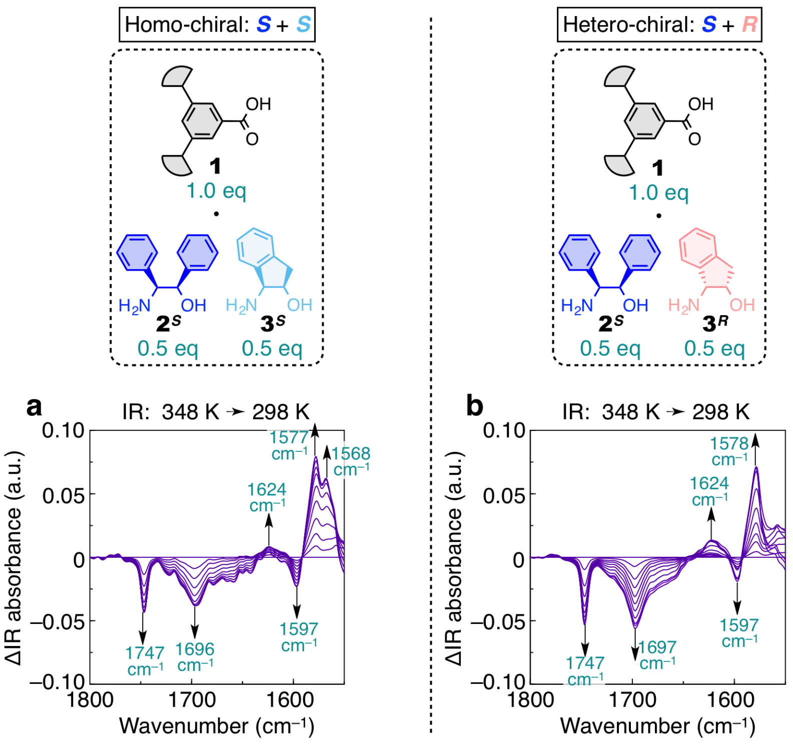

Supplementary Fig. 17 | Temperature dependent changes in FT-IR spectra of the mixtures of homo-chiral and hetero-chiral monomers.

a, Changes in FT-IR spectra at every 5 K step of a mixture of **1**•$\mathbf{2}^S$ and **1**•$\mathbf{3}^S$ ([**1**•$\mathbf{2}^S$] = [**1**•$\mathbf{3}^S$] = 2.0 mM) in dodecane measured upon cooling from 348 to 298 K at –0.5 K min^{-1}.

b, Changes in FT-IR spectra at every 5 K step of a mixture of **1**•$\mathbf{2}^S$ and **1**•$\mathbf{3}^R$ ([**1**•$\mathbf{2}^S$] = [**1**•$\mathbf{3}^R$] = 2.0 mM) in dodecane measured upon cooling from 348 to 298 K at –0.5 K min^{-1}.

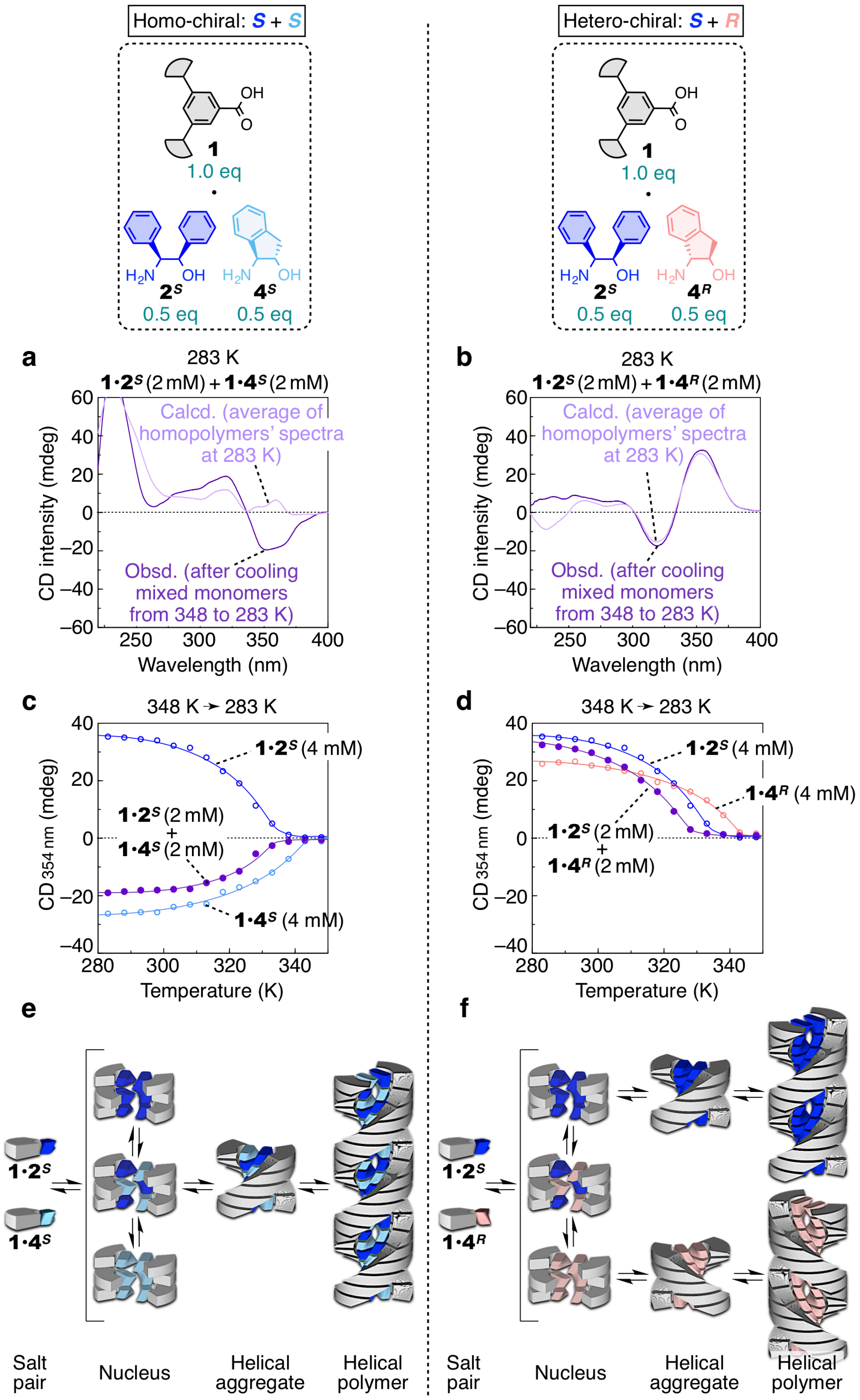

Supplementary Fig. 18 | Stereoselective supramolecular copolymerization of two monomers.

a, Obsd.: CD spectrum of a mixture of **1·2**^{S} and **1·4**^{S} in dodecane ([**1·2**^{S}] = [**1·4**^{S}] = 2.0 mM) after cooling from 348 to 283 K (–1.0 K min^{-1}).

Calcd.: Average of the CD spectra of **1·2**^{S} in dodecane (4.0 mM) and **1·4**^{S} in dodecane (4.0 mM) separately measured at 283 K.

b, Obsd.: CD spectrum of a mixture of **1•2**^{S} and **1•4**^{R} in dodecane ([**1•2**^{S}] = [**1•4**^{R}] = 2.0 mM) after cooling from 348 to 283 K (–1.0 K min^{-1}).
Calcd.: Average of the CD spectra of **1•2**^{S} in dodecane (4.0 mM) and **1•4**^{R} in dodecane (4.0 mM) separately measured at 283 K.

c, Cooling curves (from 348 to 283 K at –1.0 K min^{-1} monitored with CD intensity at 354 nm) of a mixture of **1•2**^{S} and **1•4**^{S} in dodecane ([**1•2**^{S}] = [**1•4**^{S}] = 2.0 mM), **1•2**^{S} in dodecane (4.0 mM), and **1•4**^{S} in dodecane (4.0 mM).

d, Cooling curves (from 348 to 283 K at –1.0 K min^{-1} monitored with CD intensity at 354 nm) of a mixture of **1•2**^{S} and **1•4**^{R} in dodecane ([**1•2**^{S}] = [**1•4**^{R}] = 2.0 mM), **1•2**^{S} in dodecane (4.0 mM), and **1•4**^{R} in dodecane (4.0 mM).

e,**f**, Schematic representation for the supramolecular polymerization of the homo-chiral (**1•2**^{S} and **1•4**^{S}; **e**) and hetero-chiral (**1•2**^{S} and **1•4**^{R}; **f**) mixtures.

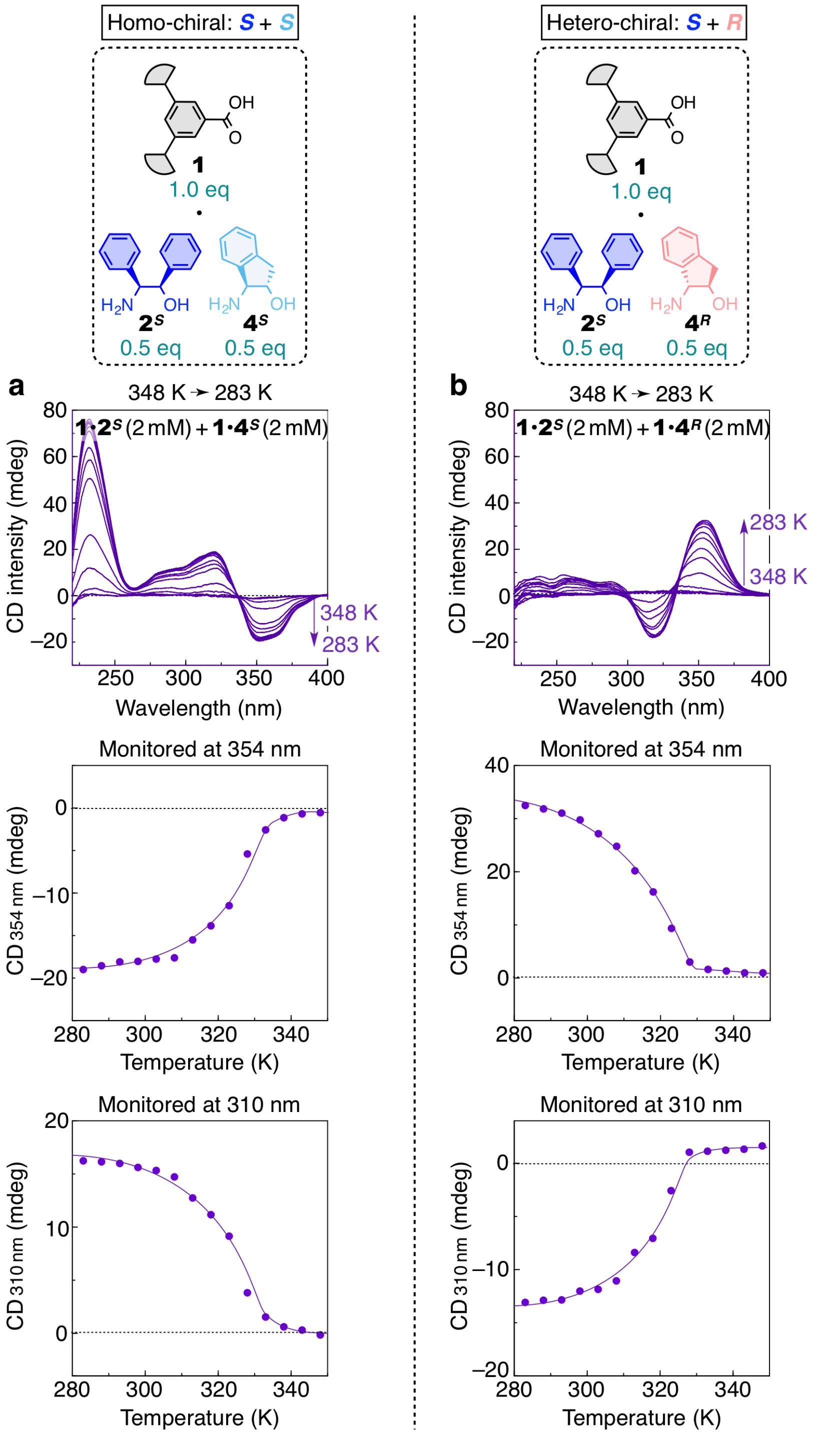

Supplementary Fig. 19 | Supramolecular copolymerization of two monomers upon cooling.

a,**b**, Variable-temperature CD profiles at every 5 K step of a mixture of **1•2**^{S} and **1•4**^{S} in dodecane ([**1•2**^{S}] = [**1•4**^{S}] = 2.0 mM; **a**) and a mixture **1•2**^{S} and **1•4**^{R} in dodecane ([**1•2**^{S}] = [**1•4**^{R}] = 2.0 mM; **b**) measured upon cooling from 348 to 283 K at –1.0 K min^{-1}.

Upper: CD spectra.

Middle and lower: Cooling curves monitored with CD intensity at 354 nm (middle) and 310 nm (lower).

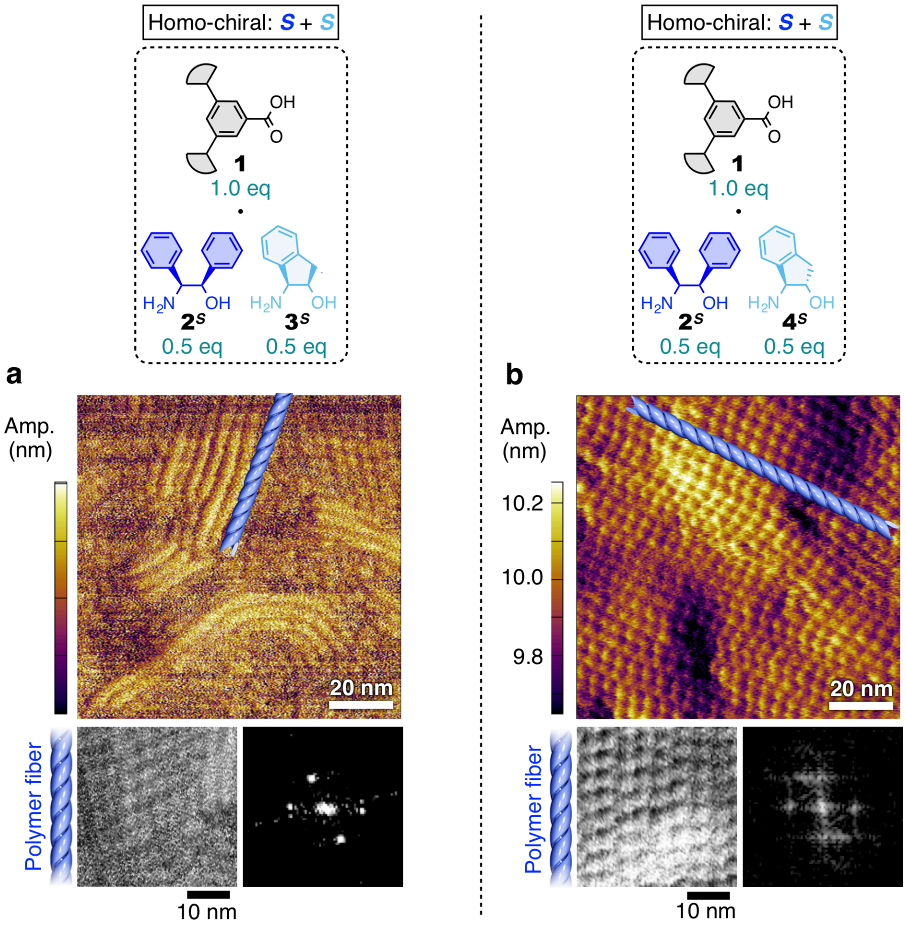

Supplementary Fig. 20 | Determination of the helical handedness of the supramolecular copolymers[8]**.**

a,**b**, AFM data of mixtures of $\mathbf{1{\cdot}2}^{S}$ and $\mathbf{1{\cdot}3}^{S}$ ([$\mathbf{1{\cdot}2}^{S}$] = [$\mathbf{1{\cdot}3}^{S}$] = 2.0 mM; **a**) and $\mathbf{1{\cdot}2}^{S}$ and $\mathbf{1{\cdot}4}^{S}$ ([$\mathbf{1{\cdot}2}^{S}$] = [$\mathbf{1{\cdot}4}^{S}$] = 2.0 mM; **b**) spin-coated on HOPG substrates from dodecane solutions.

Upper: AFM amplitude images.

Lower left: Grey-scale images (38 nm × 38 nm) trimmed from the AFM images.

Lower right: Magnified FFT images of the grey-scale images.

The illustrations of helices indicate the orientation direction of the supramolecular polymer fibres. For details of FFT, see Supplementary Methods.

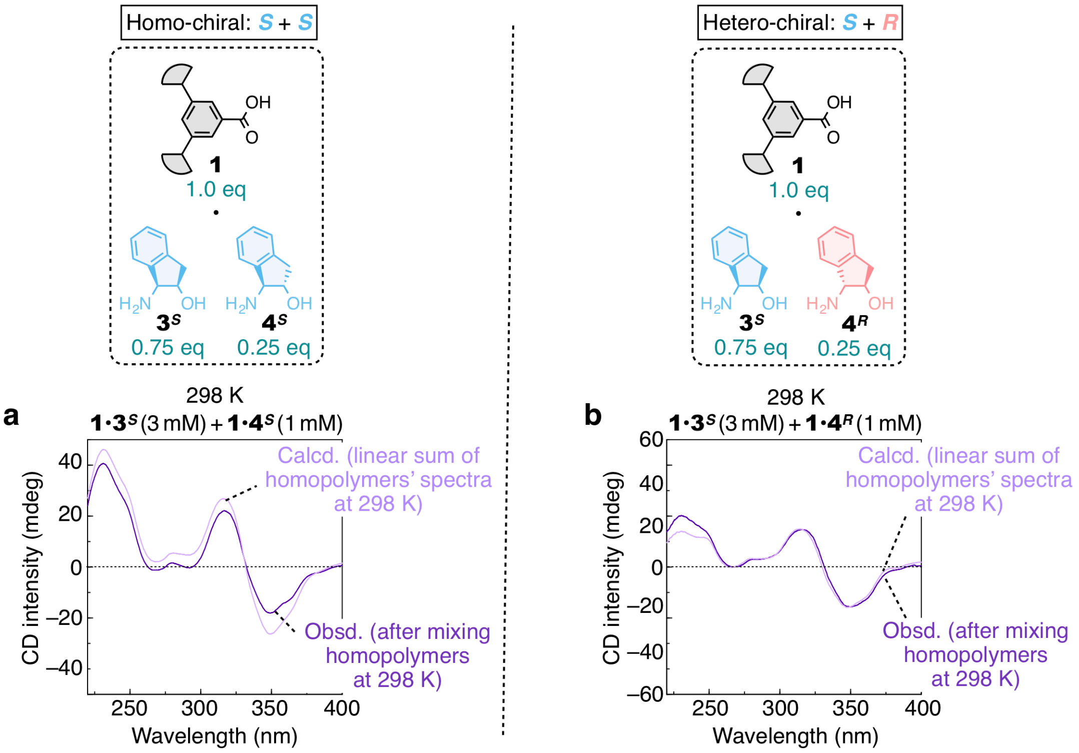

Supplementary Fig. 21 | Stereoselective supramolecular copolymerization of two monomers.

a, Obsd.: CD spectrum of a mixture of **1•3**^{S} and **1•4**^{S} in dodecane ([**1•3**^{S}] = 3.0 mM; [**1•4**^{S}] = 1.0 mM) at 298 K.

Calcd.: Linear sum of CD spectra of **1•3**^{S} in dodecane (3.0 mM) and **1•4**^{S} in dodecane (1.0 mM) separately measured at 298 K.

b, Obsd.: CD spectrum of a mixture of **1•3**^{S} and **1•4**^{R} in dodecane ([**1•3**^{S}] = 3.0 mM; [**1•4**^{R}] = 1.0 mM) at 298 K.

Calcd.: Linear sum of the CD spectra of **1•3**^{S} in dodecane (3.0 mM) and **1•4**^{R} in dodecane (1.0 mM) separately measured at 298 K.

The mixtures of **1•3**^{S} and **1•4**^{S} in dodecane were highly prone to turn into gel or form precipitates, probably because of the bundling of the supramolecular polymer chains. To keep the homogeneity of the samples and to avoid the effects of linear dichroism, the present mixing experiments were done in the above mixing ratio and at 298 K.

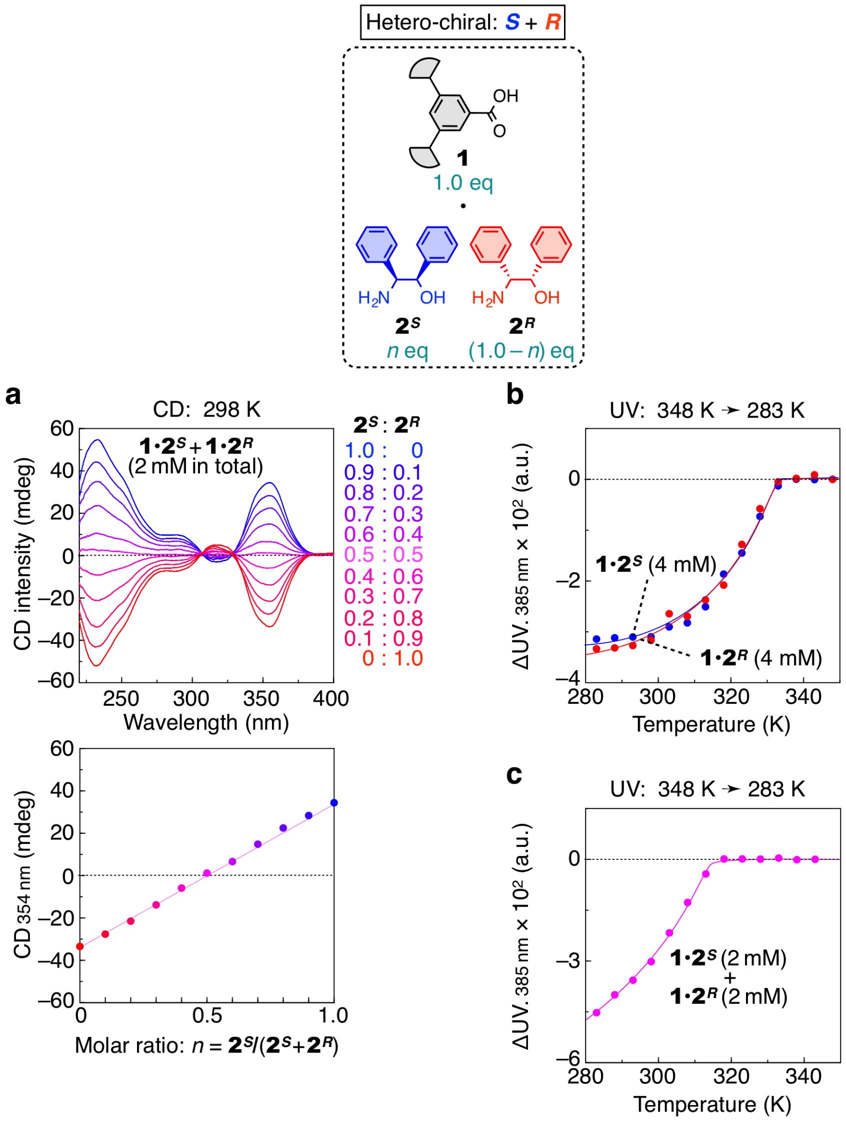

Supplementary Fig. 22 | Stereoselective supramolecular copolymerization of enantiomeric monomers.

a, CD profiles of mixtures of **1•2**^{S} and **1•2**^{R} with various ratios in dodecane (2.0 mM in total).

Upper: CD spectra.

Lower: Plot of CD intensity at 354 nm against the molar ratio of **2**^{S}. The data points for enantiopure **1•2**^{S} and **1•2**^{R} are connected with a straight line.

b,**c**, Cooling curves (from 348 to 283 K at –1.0 K min^{-1}, monitored with UV absorption at 385 nm) of enantiopure **1•2**^{S} in dodecane (4.0 mM; **b**, blue), enantiopure **1•2**^{R} in dodecane (4.0 mM; **b**, red), and a racemic mixture of **1•2**^{S} and **1•2**^{R} in dodecane ([**1•2**^{S}] = [**1•2**^{R}] = 2.0 mM; **c**).

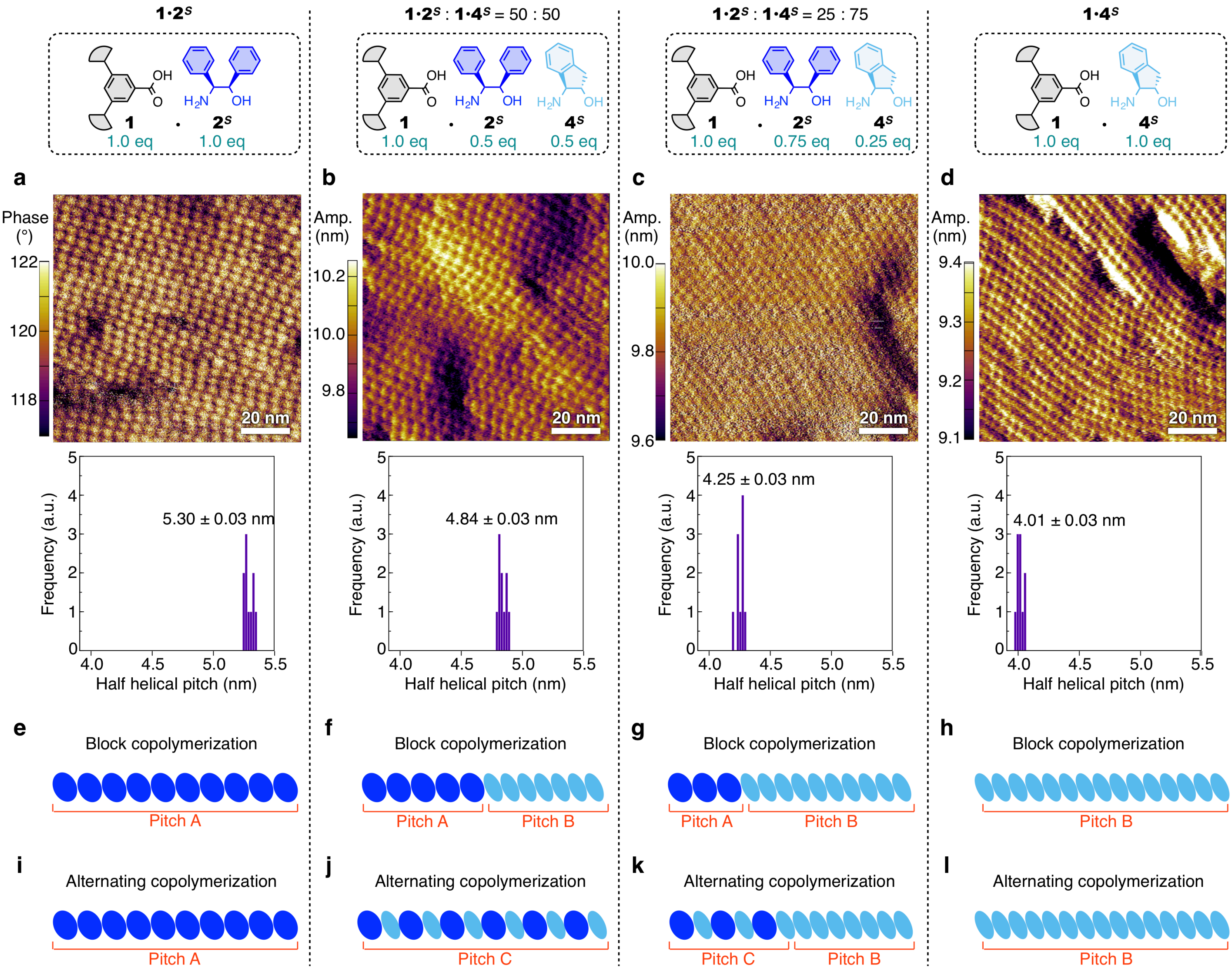

Supplementary Fig. 23 | Investigation of copolymerization mode from the AFM images of mixtures with various monomer ratios.

a–**d**, AFM data of mixtures of **1•2**^{S} and **1•4**^{S} with various molar ratios: **1•2**^{S}:**1•4**^{S} = 100:0 (**a**), 50:50 (**b**), 25:75 (**c**), and 0:100 (**d**). The mixtures were spin-coated on HOPG substrates from their dodecane solutions ([**1•2**^{S}] + [**1•4**^{S}] = 4.0 mM).

Upper: AFM phase/amplitude images.

Lower: Histograms of the half helical pitches of the fibres in the AFM images. Ten fibres were randomly selected from each AFM image. The half helical pitch was determined as the average of those of five consecutive turns.

e–**l**, Supposed structures formed by the block (**e**–**h**) and alternating (**i**–**l**) copolymerization of **1•2**^{S} and **1•4**^{S} with various molar ratios: **1•2**^{S}:**1•4**^{S} = 100:0 (**e**,**i**), 50:50 (**f**,**j**), 25:75 (**g**,**k**), and 0:100 (**h**,**l**).

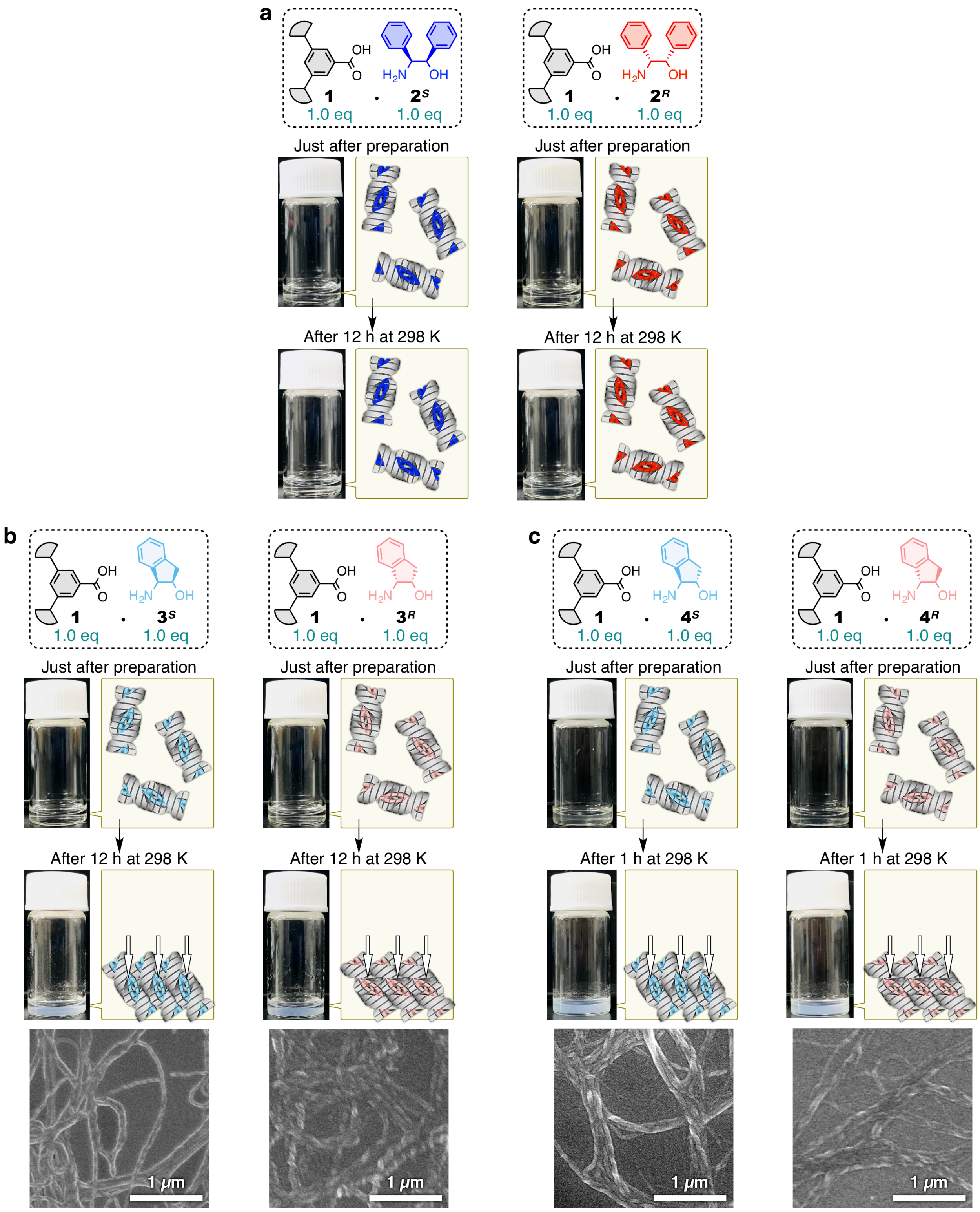

Supplementary Fig. 24 | Solution stability of the helical supramolecular polymers.

a–**c**, Photographs and schematics of **1•2**^{S}/**1•2**^{R} (**a**), **1•3**^{S}/**1•3**^{R} (**b**) and **1•4**^{S}/**1•4**^{R} (**c**) in dodecane (4.0 mM, 400 μL) at 298 K.

Upper: Just after preparation.

Lower: After incubation at 298 K. SEM images of the precipitates are shown for **b** and **c**.

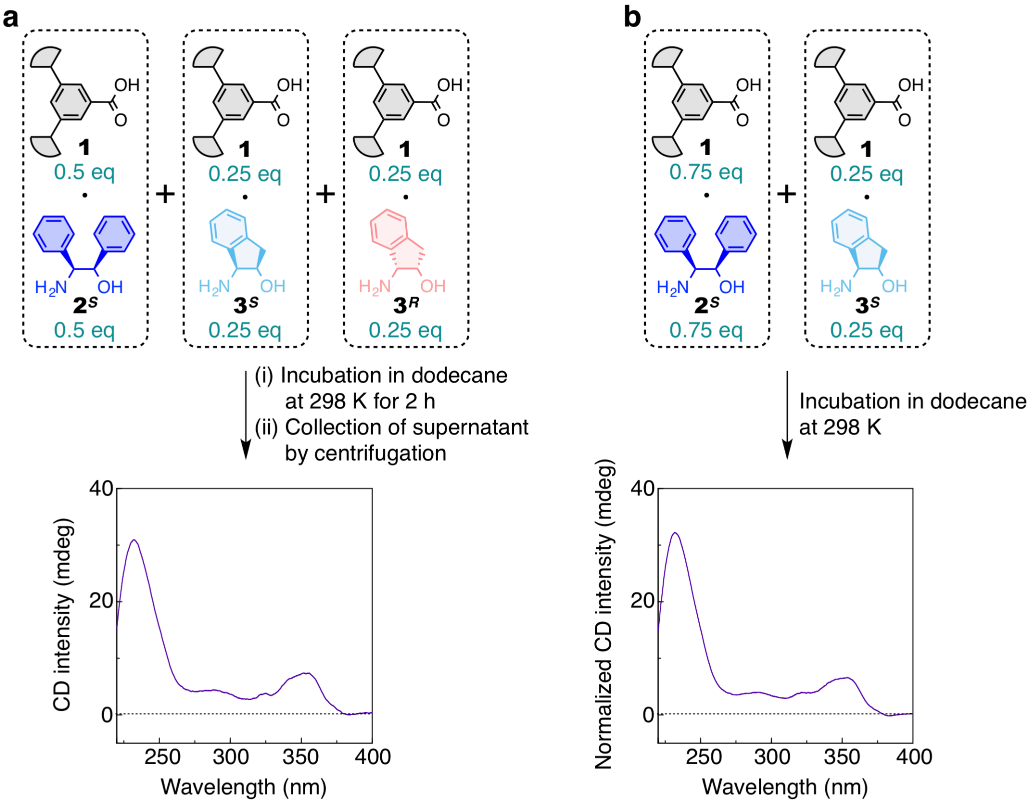

Supplementary Fig. 25 | CD spectral characterization of the supernatant obtained through the enantio-separation in Fig. 6c.

a, CD spectrum at 298 K of the supernatant obtained through the enantio-separation in Fig. 6c.

b, CD spectrum at 298 K of a mixture of **1•2**^{S} and **1•3**^{S} in dodecane ([**1•2**^{S}] = 1.88 mM; [**1•3**^{S}] = 0.63 mM).

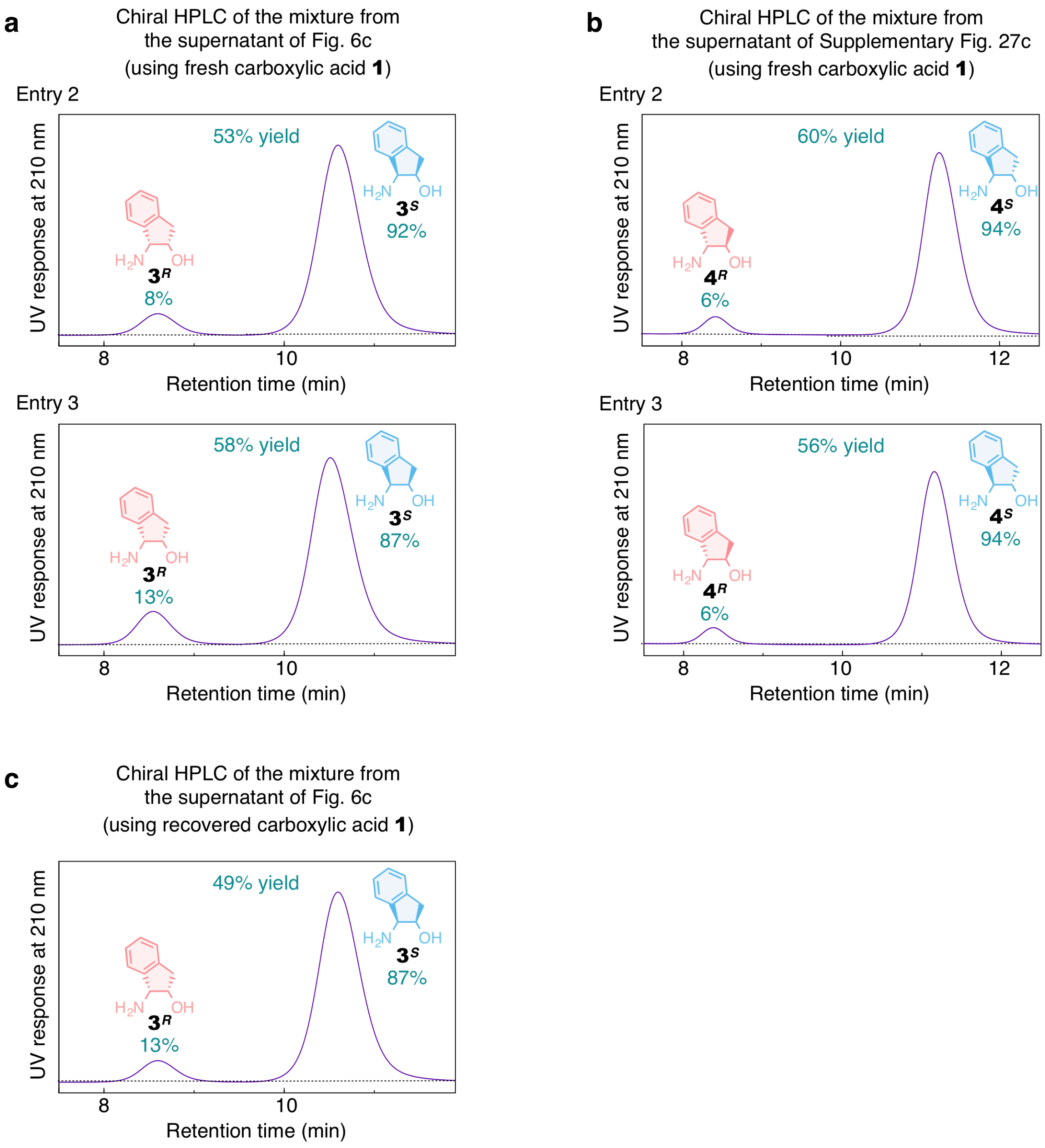

Supplementary Fig. 26 | Reproducibility of the chiral separation of amino alcohols by the helical supramolecular polymer.

a, Chiral HPLC traces of the mixture of $\mathbf{3}^{S}$ and $\mathbf{3}^{R}$ obtained from the supernatant of Fig. 6c. The same procedure was repeated.

b, Chiral HPLC traces of the mixture of $\mathbf{4}^{S}$ and $\mathbf{4}^{R}$ obtained from the supernatant of Supplementary Fig. 27c. The same procedure was repeated.

c, Chiral HPLC traces of the mixture of $\mathbf{3}^{S}$ and $\mathbf{3}^{R}$ obtained from the supernatant of Fig. 6c where the carboxylic acid **1** recovered from the supernatant and precipitate in the first cycle of the enantio-separation of Fig. 6c was used.

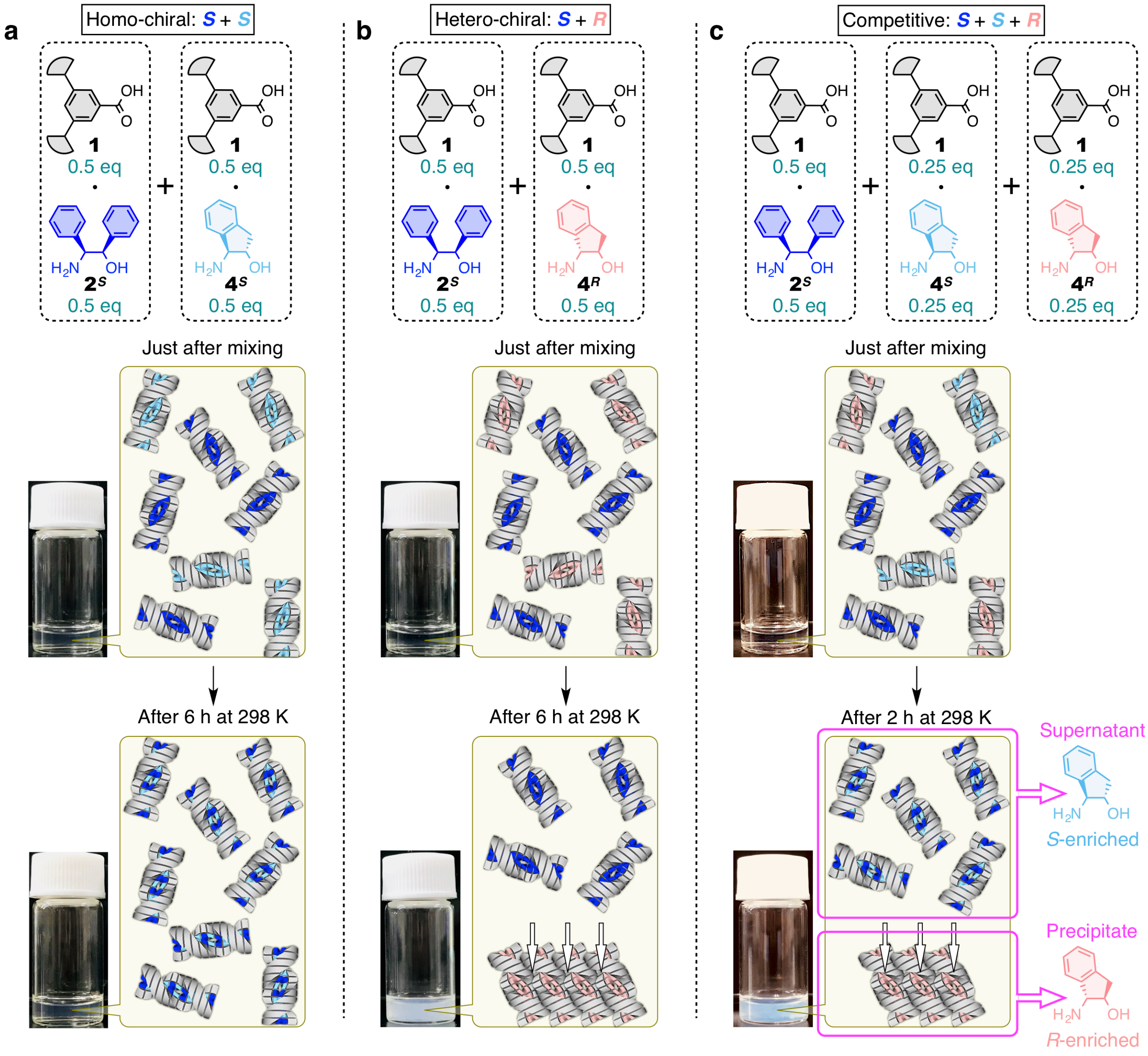

Supplementary Fig. 27 | Enantio-separation of amino alcohols by the helical supramolecular polymer: recognition and separation of 1•4^{S}/1•4^{R} by 1•2^{S}.

a–c, Photographs and schematic representations of samples prepared at 298 K by mixing separately prepared dodecane solutions (4.0 mM) of **1•2^{S}** (400 μL) and **1•4^{S}** (400 μL) (**a**), **1•2^{S}** (400 μL) and **1•4^{R}** (400 μL) (**b**), and **1•2^{S}** (400 μL), **1•4^{S}** (200 μL) and **1•4^{R}** (200 μL) (**c**).

Upper: Just after mixing. Lower: After incubation at 298 K.

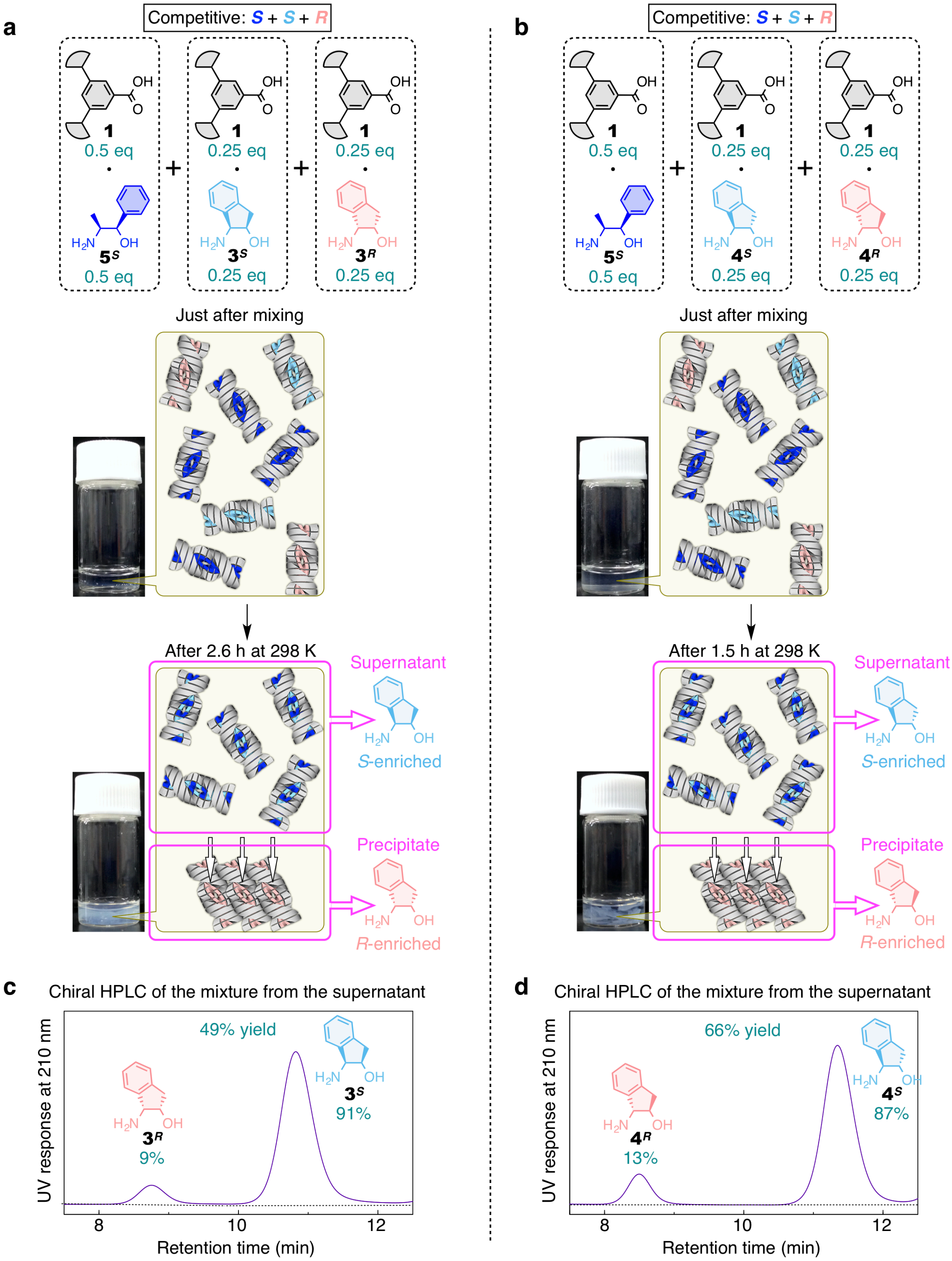

Supplementary Fig. 28 | Enantio-separation of amino alcohols by the helical supramolecular polymer: separation of 1·3^S/1·3^R and 1·4^S/1·4^R by 1·5^S.

a,**b**, Photographs and schematic representations of samples prepared at 298 K by mixing separately prepared dodecane solutions of **1•5**^{S} (4.0 mM, 400 μL), **1•3**^{S} (4.0 mM, 200 μL) and **1•3**^{R} (4.0 mM, 200 μL) (**a**) and **1•5**^{S} (2.0 mM, 800 μL), **1•4**^{S} (4.0 mM, 200 μL) and **1•4**^{R} (4.0 mM, 200 μL) (**b**).
Upper: Just after mixing. Lower: After incubation at 298 K.

c, Chiral HPLC trace of the mixture of **3**^{S} and **3**^{R} obtained from the supernatant of **a**.

d, Chiral HPLC trace of the mixture of **4**^{S} and **4**^{R} obtained from the supernatant of **b**.

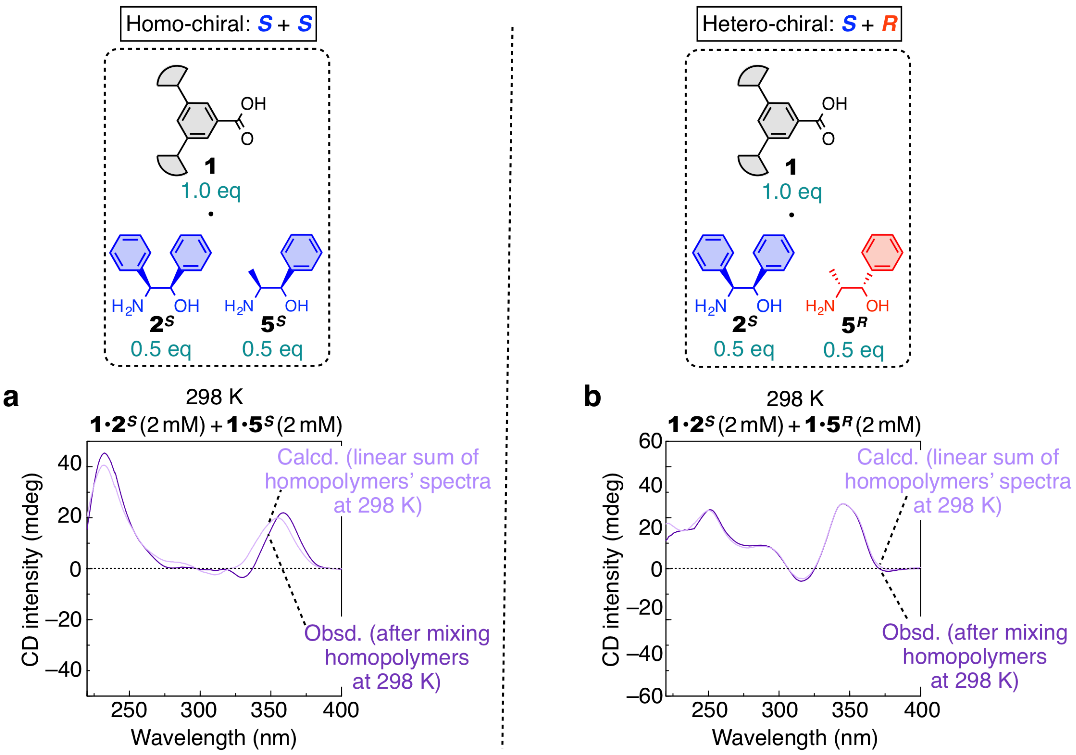

Supplementary Fig. 29 | Stereoselective supramolecular copolymerization of two monomers.

a, Obsd.: CD spectrum of a mixture of **1**•**2**^{S} and **1**•**5**^{S} in dodecane ([**1**•**2**^{S}] = [**1**•**5**^{S}] = 2.0 mM) at 298 K. Calcd.: Average of the CD spectra of **1**•**2**^{S} in dodecane (4.0 mM) and **1**•**5**^{S} in dodecane (4.0 mM) separately measured at 298 K.

b, Obsd.: CD spectrum of a mixture of **1**•**2**^{S} and **1**•**5**^{R} in dodecane ([**1**•**2**^{S}] = [**1**•**5**^{R}] = 2.0 mM) at 298 K. Calcd.: Average of the CD spectra of **1**•**2**^{S} in dodecane (4.0 mM) and **1**•**5**^{R} in dodecane (4.0 mM) separately measured at 298 K.

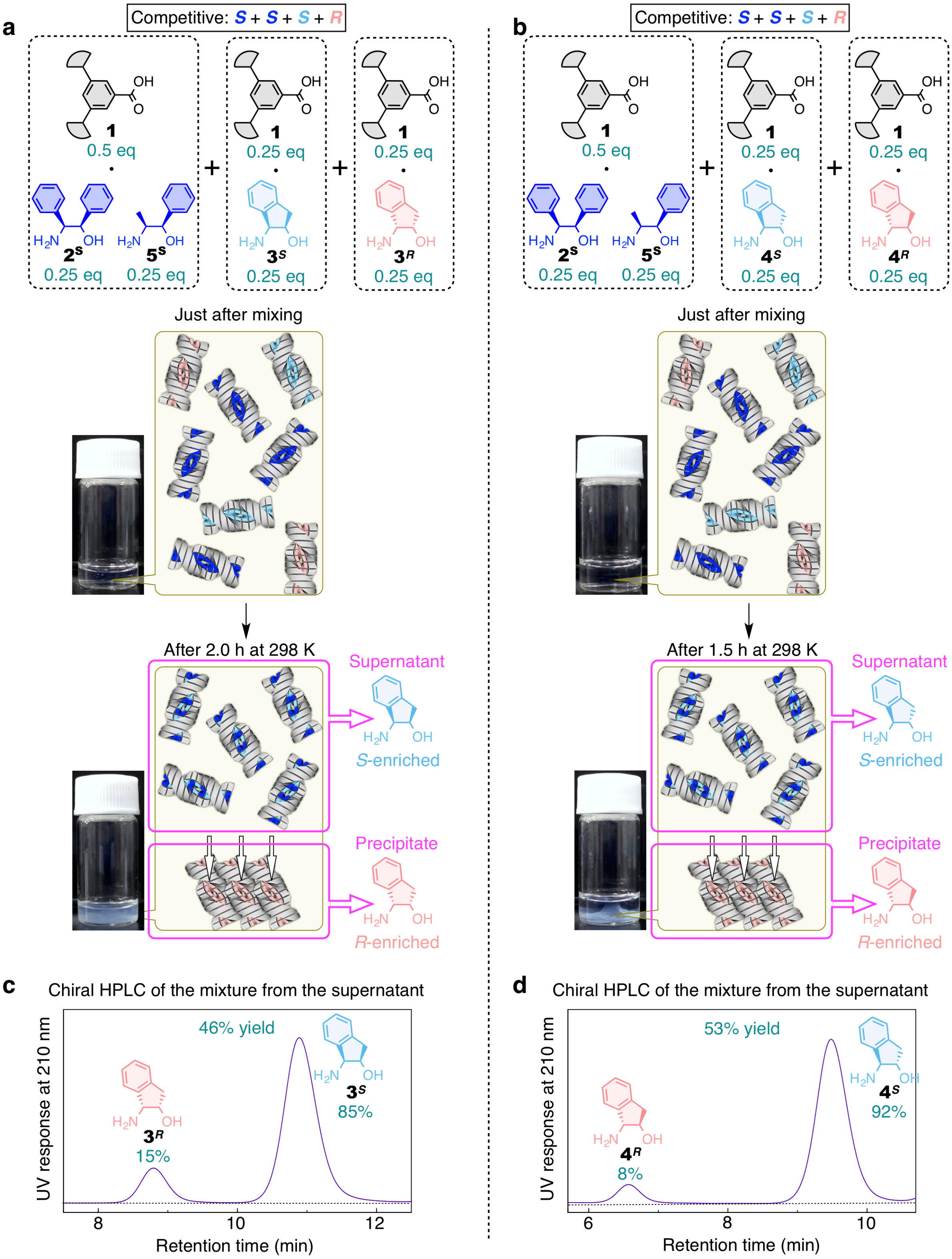

Supplementary Fig. 30 | Enantio-separation of amino alcohols by the helical supramolecular copolymer: separation of $1{\cdot}3^S/1{\cdot}3^R$ and $1{\cdot}4^S/1{\cdot}4^R$ by a mixture of $1{\cdot}2^S$ and $1{\cdot}5^S$

a,**b**, Photographs and schematic representations of samples prepared at 298 K by mixing separately prepared dodecane solutions of a mixture of **1•2**^{S} and **1•5**^{S} ([**1•2**^{S}] = [**1•5**^{S}] = 2.0 mM; 400 μL), **1•3**^{S} (4.0 mM; 200 μL) and **1•3**^{R} (4.0 mM; 200 μL) (**a**) and a mixture of **1•2**^{S} and **1•5**^{S} ([**1•2**^{S}] = [**1•5**^{S}] = 1.0 mM; 800 μL), **1•4**^{S} (4,0 mM; 200 μL) and **1•4**^{R} (4.0 mM; 200 μL) (**b**).
Upper: Just after mixing. Lower: After incubation at 298 K.

c, Chiral HPLC trace of the mixture of **3**^{S} and **3**^{R} obtained from the supernatant of **a**.

d, Chiral HPLC trace of the mixture of **4**^{S} and **4**^{R} obtained from the supernatant of **b**.

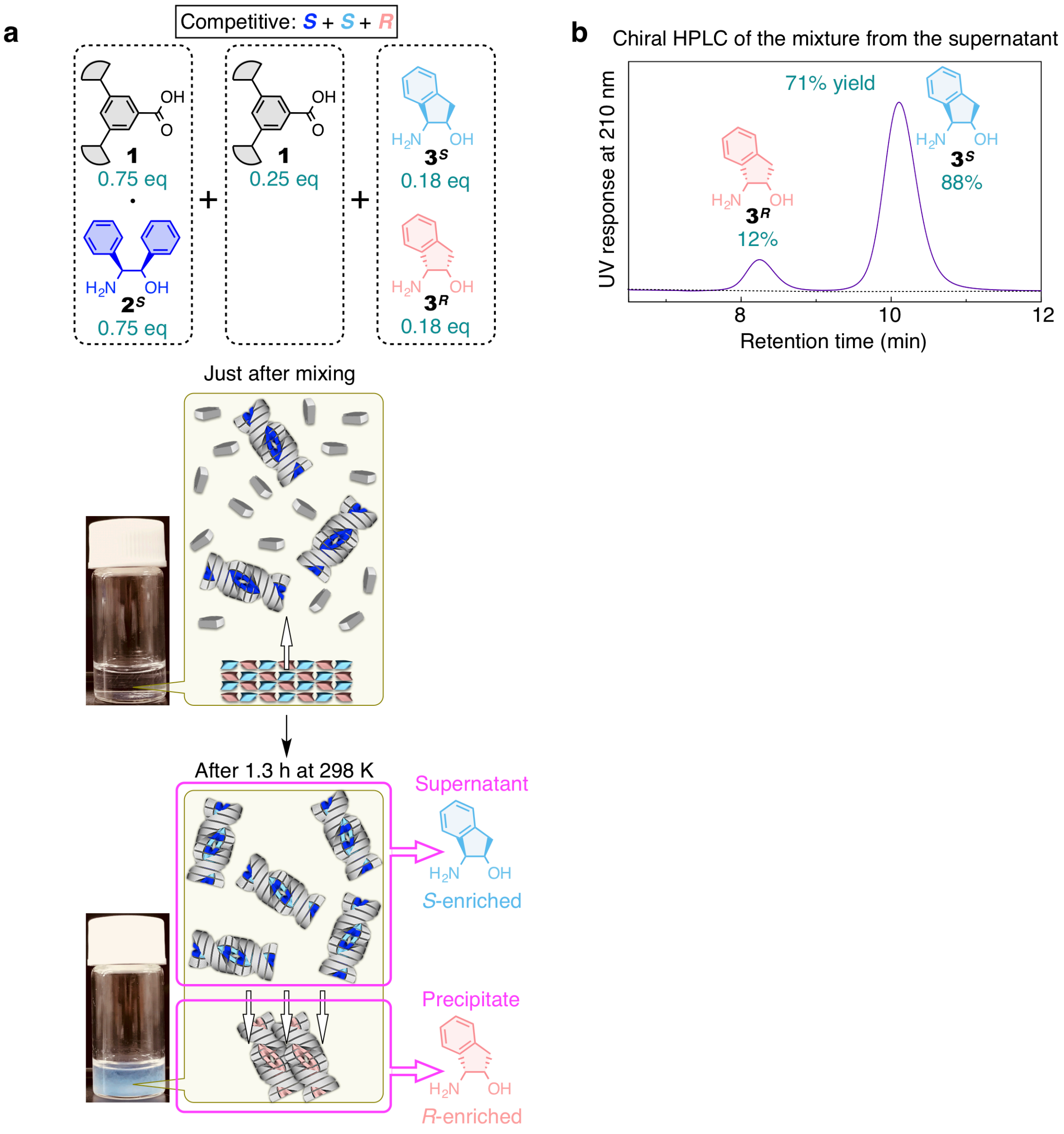

Supplementary Fig. 31 | Enantio-separation of amino alcohols by the helical supramolecular polymer: direct enantio separation of racemic $3^S/3^R$ by $1{\cdot}2^S$.

a, Photographs and schematic representations of a sample prepared at 298 K by mixing a dodecane solution (1,200 μL) containing $\mathbf{1{\cdot}2}^S$ (3.0 mM; 3.60 μmol) and free acid **1** (1.0 mM; 1.20 μmol) was mixed with a racemic solid of $\mathbf{3}^S/\mathbf{3}^R$ (0.87 μmol/0.87 μmol).

Upper: Just after mixing. Lower: After incubation at 298 K.

b, Chiral HPLC trace of the mixture of $\mathbf{3}^S$ and $\mathbf{3}^R$ obtained from the supernatant of **a**.

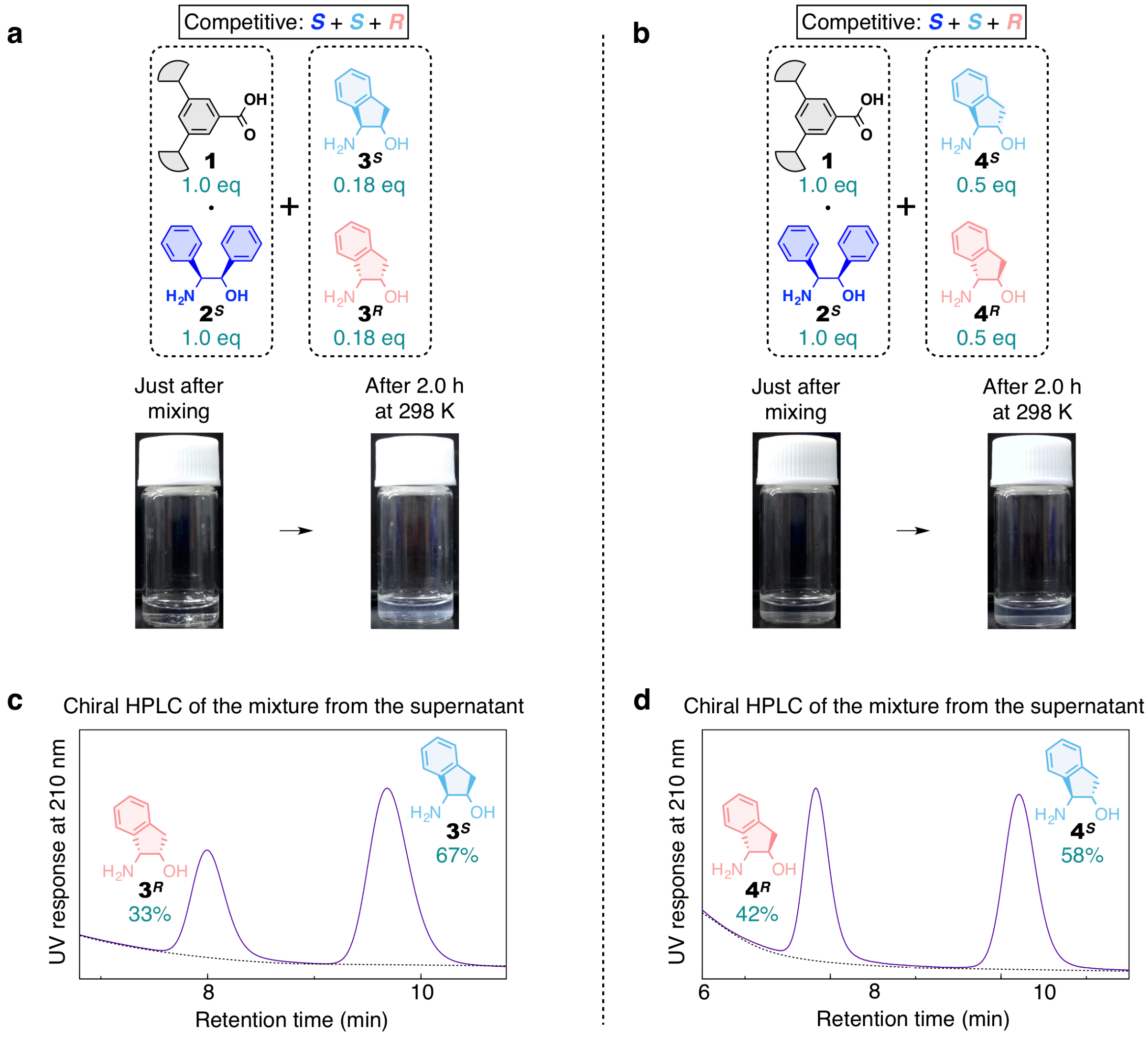

Supplementary Fig. 32 | Enantio-separation of amino alcohols by the helical supramolecular polymer: direct enantio separation of racemic 3^S/3^R and 4^S/4^R by 1•2^S without free acid 1.

a, Photographs of a sample prepared at 298 K by mixing a dodecane solution (400 μL) containing **1**•$\mathbf{2}^S$ (4.0 mM; 1.60 μmol) was mixed with a racemic solid of $\mathbf{3}^S$/$\mathbf{3}^R$ (0.80 μmol/0.80 μmol). Left: Just after mixing. Right: After incubation at 298 K.

b, Photographs of a sample prepared at 298 K by mixing a dodecane solution (400 μL) containing **1**•$\mathbf{2}^S$ (4.0 mM; 1.60 μmol) was mixed with a racemic solid of $\mathbf{4}^S$/$\mathbf{4}^R$ (0.80 μmol/0.80 μmol). Left: Just after mixing. Right: After incubation at 298 K.

c, Chiral HPLC trace of the mixture of $\mathbf{3}^S$ and $\mathbf{3}^R$ obtained from the supernatant of **a**.

d, Chiral HPLC trace of the mixture of $\mathbf{4}^S$ and $\mathbf{4}^R$ obtained from the supernatant of **b**.

Supplementary References

1. Arendt, M., Sun, W., Thomann, J., Xie, X. & Schrader, T. Dendrimeric bisphosphonates for multivalent protein surface binding. *Chem. Asian J.* **1**, 544–554 (2006).
2. Fischer, G. M., Daltrozzo, E. & Zumbusch, A. Selective NIR chromophores: Bis(pyrrolopyrrole) cyanines. *Angew. Chem. Int. Ed.* **50**, 1406–1409 (2011).
3. Groeper, J. A., Hitchcock, S. R. & Ferrence, G. M. A scalable and expedient method of preparing diastereomerically and enantiomerically enriched pseudonorephedrine from norephedrine. *Tetrahedron: Asymmetry* **17**, 2884–2889 (2006).
4. Zhou, L., Wang, Z., Wei, S & Sun, J. Evolution of chiral Lewis basic N-formamide as highly effective organocatalyst for asymmetric reduction of both ketones and ketimines with an unprecedented substrate scope. *Chem. Commun.* **28***,* 2977–2979 (2007).
5. Banerjee, S., Camodeca, A. J., Griffin, G. G., Hamaker, C. G., & Hitchcock, S. R. Aromatic motifs in the design of Ephedra ligands for application in the asymmetric addition of diethylzinc to aldehydes and diphenylphosphinoylimines. *Tetrahedron: Asymmetry* **21**, 549–557 (2010).
6. Duraimurugan, K. & Siva, A. Phenylene(vinylene) based fluorescent polymer for selective and sensitive detection of nitro-explosive picric acid. *J. Polym. Sci., Part A: Polym. Chem.* **54**, 3800–3807 (2016).
7. Fujisawa, T. *et al.* Small-angle X-ray scattering station at the SPring-8 RIKEN beamline. *J. Appl. Crystallogr.* **33**, 797–800 (2000).
8. Sakurai, S., Okoshi, K., Kumaki, J. & Yashima, E. Two-dimensional hierarchical self-assembly of one-handed helical polymers on graphite. *Angew. Chem. Int. Ed.* **45**, 1245–1248 (2006).
9. http://imagej.nih.gov/ij/
10. Kinbara, K., Kobayashi, Y. & Saigo, K. Systematic study of chiral discrimination upon crystallisation. Part 2. Chiral discrimination of 2-arylalkanoic acids by (1*R*,2*S*)-2-amino-1,2-diphenylethanol. *J. Chem. Soc., Perkin Trans. 2* 1767–1775 (1998).
11. Kodama, K., Kobayashi, Y. & Saigo, K. Two-component supramolecular helical architectures: creation of tunable dissymmetric cavities for the inclusion and chiral recognition of the third components. *Chem. Eur. J.* **13**, 2144–2152 (2007).
12. Li, C. *et al.* Macroscopic ordering of helical pores for arraying guest molecules noncentrosymmetrically. *Nat. Commun.* **6**, 8418 (2015).
